# Artificial intelligence-based comprehensive analysis of immune-stemness-tumor budding profile to predict survival of patients with pancreatic adenocarcinoma

**DOI:** 10.20892/j.issn.2095-3941.2022.0569

**Published:** 2023-03-24

**Authors:** Tianxing Zhou, Quan Man, Xueyang Li, Yongjie Xie, Xupeng Hou, Hailong Wang, Jingrui Yan, Xueqing Wei, Weiwei Bai, Ziyun Liu, Jing Liu, Jihui Hao

**Affiliations:** 1 Department of Pancreatic Cancer, Tianjin Medical University Cancer Institute & Hospital, National Clinical Research Center for Cancer, Key Laboratory of Cancer Prevention and Therapy, Tianjin, Tianjin’s Clinical Research Center for Cancer, Tianjin 300060, China; 2 Department of Hepatopancreatobiliary Surgery, Tongliao City Hospital, Tongliao 028000, China; 3 Department of Breast Oncoplastic Surgery; 4 Department of Cancer Cell Biology; 5 Department of Diagnostic and Therapeutic Ultrasonography, Tianjin Medical University Cancer Institute & Hospital, National Clinical Research Center for Cancer, Key Laboratory of Cancer Prevention and Therapy, Tianjin, Tianjin’s Clinical Research Center for Cancer, Key Laboratory of Breast Cancer Prevention and Therapy, Tianjin Medical University, Ministry of Education, Tianjin 300060, China

**Keywords:** Artificial intelligence, CD8, CSCs, tumor budding, PDAC, nomogram

## Abstract

**Objective:**

Pancreatic ductal adenocarcinoma (PDAC) is an aggressive malignancy. CD8^+^ T cells, cancer stem cells (CSCs), and tumor budding (TB) have been significantly correlated with the outcome of patients with PDAC, but the correlations have been independently reported. In addition, no integrated immune-CSC-TB profile for predicting survival in patients with PDAC has been established.

**Methods:**

Multiplexed immunofluorescence and artificial intelligence (AI)-based comprehensive analyses were used for quantification and spatial distribution analysis of CD8^+^ T cells, CD133^+^ CSCs, and TB. *In vivo* humanized patient-derived xenograft (PDX) models were established. Nomogram analysis, calibration curve, time-dependent receiver operating characteristic curve, and decision curve analyses were performed using R software.

**Results:**

The established ‘anti-/pro-tumor’ models showed that the CD8^+^ T cell/TB, CD8^+^ T cell/CD133^+^ CSC, TB-adjacent CD8^+^ T cell, and CD133^+^ CSC-adjacent CD8^+^ T cell indices were positively associated with survival of patients with PDAC. These findings were validated using PDX-transplanted humanized mouse models. An integrated nomogram-based immune-CSC-TB profile that included the CD8^+^ T cell/TB and CD8^+^ T cell/CD133^+^ CSC indices was established and shown to be superior to the tumor-node-metastasis stage model in predicting survival of patients with PDAC.

**Conclusions:**

‘Anti-/pro-tumor’ models and the spatial relationship among CD8^+^ T cells, CSCs, and TB within the tumor microenvironment were investigated. Novel strategies to predict the prognosis of patients with PDAC were established using AI-based comprehensive analysis and machine learning workflow. The nomogram-based immune-CSC-TB profile can provide accurate prognosis prediction for patients with PDAC.

## Introduction

Pancreatic ductal adenocarcinoma (PDAC) is an aggressive malignancy^1^ that is projected to become the second most lethal tumor by 2030^2^. Currently, the tumor-node-metastasis (TNM) classification system is widely used for PDAC staging^3^; however, the clinical outcome may significantly vary among patients with the same TNM stage. Therefore, researchers are intensively searching for additional factors that are highly related to prognosis.

The tumor microenvironment (TME) is a complex assembly of genetically heterogeneous cancer cells and different cell types that constitute the local environment^4,5^. These cells include endothelial cells, cancer-associated fibroblasts, and different populations of immune cells. The development of PDAC is closely associated with the TME^6^. Cytotoxic CD8^+^ T cells, regulatory T cells (Tregs), myeloid-derived suppressor cells (MDSCs), mast cells, tumor-associated macrophages (TAMs), tumor-associated neutrophils (TANs), cancer stem cells (CSCs), and cancer-associated fibroblasts (CAFs) could account for PDAC progression and survival of PDAC patients^7–14^; however, the interactions among these factors in PDAC remain unclear.

Investigations on tumor immunity and host defense in patients with PDAC demonstrate promising results for immunotherapy^15^. Tumor-infiltrated lymphocytes (TILs), especially CD8^+^ cytotoxic T cells, are significantly related to overall- and disease-specific survival in patients with PDAC^16^. CSCs are a subset of cells exhibiting self-renewal, multi-differentiation, and tumor-initiating capacities^17^. Several studies have reported that CSCs contribute to radio- and chemo-therapy resistance, tumor metastases, and tumor progression in patients with PDAC. A high-stemness signature has been correlated with a poor immunogenic response in many solid malignancies, highlighting the potential interaction between tumor immune and CSC profiles and indicating that CSCs may acquire the capacity to evade the host immune response^7,18,19^. Tumor budding (TB) of single tumor cells or tumor cell clusters with up to five cells from the invasive tumor front represents a highly invasive tumor subpopulation^20^. TB has been associated with lymph node positivity, poorly differentiated tumors, vascular and lymphatic invasion, local tumor recurrence, and distant metastases. Karamitopoulou et al.^21^ reported that high-grade TB can be used as a powerful, independent, and highly unfavorable prognostic factor in patients with PDAC. Although tumor-infiltrated CD8^+^ T lymphocytes, CSCs, and TB are all related to prognosis of patients with PDAC, no studies evaluating tumor-infiltrated CD8^+^ T lymphocytes, CSCs, and TB together have been reported. Therefore, we established an ‘anti-/pro-tumor model’ defined by an established host-related ‘anti-tumor’ factor (CD8^+^ T lymphocytes) and ‘pro-tumor’ factors (CSCs and TB), and evaluated the prognostic significance of the ‘anti-/pro-tumor model’ in patients with PDAC.

Previous studies used an automatic imaging and scoring platform to quantify and analyze tumor-infiltrated CD8^+^ T lymphocytes, CD133^+^ CSCs, and TB within the TME to achieve a greater level of standardization than subjective, manual reporting^11,22,23^; however, these cells have yet to be studied together in patients with PDAC in an ‘anti-/pro-tumor model’ using an automatic quantification and analysis approach. Moreover, the spatial interaction among the components within the TME could be studied using the same tissue section.

The TME, including CD8^+^ T cells, CSCs, and TB, is vital for predicting prognosis of patients with PDAC. By scoring different prognostic factors for each individual patient, a nomogram-based survival prediction model has shown superiority in predicting prognosis^24^. As a visual representation of the hazard ratios of multiple prognostic factors, a nomogram can provide accurate clues to quantify each factor in the final comprehensive profiles. In our study a nomogram-based model, including the CD8^+^ T cell/CD133^+^ CSC and CD8^+^ T cell/TB indices, was further constructed and validated. This prediction model showed superior survival predictability than the TNM stage model in patients with PDAC.

## Materials and methods

Detailed information of methods and reagents are also described in the **Supplementary Methods** and **Table S8**.

### Study design and patient cohort

This study was performed on two independent retrospective cohorts and one prospective cohort of patients with PDAC. The inclusion criteria were as follows: no history of other malignancies; no neo-adjuvant chemotherapy or radiotherapy; no preoperative unrespectable tumors or distant metastases; pathologically-verified PDAC; complete clinical and pathologic data; 100% follow-up information; and systemic gemcitabine-based chemotherapy. The exclusion criteria were perioperative mortality and development of a second primary cancer during follow-up. The retrospective primary training cohort included 160 patients with PDAC who received care at the Tianjin Medical University Cancer Institute & Hospital (Tianjin, China) from July 2011 to January 2015. One hundred eight patients who received care at the Tianjin Medical University Cancer Institute & Hospital from July 2016 to January 2018 were assigned to another retrospective validation cohort. The clinical characteristics of the two independent retrospective and prospective cohorts of patients with PDAC are listed in **Supplementary Table S1**. The prospective validation cohort included 63 patients with PDAC who received care at the Tianjin Medical University Cancer Institute & Hospital from January 2016 to January 2017 (**Supplementary Table S2**). Furthermore, another retrospective validated cohort including 95 PDAC patients from Department of Hepatopancreatobiliary Surgery, Tongliao City Hospital (Tongliao, Inner Mongolia, China) were also recruited. The clinical characteristics of this retrospective cohort of patients with PDAC are listed in **Supplementary Table S3**.

All patients were categorized in accordance with the National Comprehensive Cancer Network (NCCN) TNM staging system. Use of specimens and patient information was approved by the Ethics Committees of the Tianjin Medical University Cancer Institute & Hospital and Tongliao City Hospital. All patients provided written consent for the use of their specimens and disease information for future investigations in accordance with the Ethics Committee approval and the Declaration of Helsinki (Approval Nos. AE-2021021 and 2021024).

### Immunohistochemistry (IHC), hematoxylin and eosin (H&E) staining, image capture, and scoring

IHC assays for CD8 (ZA-0508; ZSGB-BIO, City of Beijing, China), CD133 (ab226355; Abcam, City of Cambridge, USA), CK19 (ab7755; Abcam) PD-1 (ab52587; Abcam), and Tim-3 (ab241332; Abcam) were performed on pancreatic cancer tissues using and IHC kit (ZLI-9018; ZSGB-BIO, City of Beijing, China) in accordance with standard protocols following a previously described procedure^25^.

### Multiplexed immunofluorescence

Multiplexed immunofluorescence assays for CD8, CD133 and CK19, PD-1, and Tim-3 were performed on pancreatic cancer tissues as previous described^25^.

### Artificial intelligence (AI)-based image analysis

Digital whole slide fluorescence images were uploaded into Tissue Gnostics Image Analysis software (version 2.4; Zeiss, City of Oberkochen, Germany) for image analysis. The full algorithm workflow and settings are shown below.

### Cellular segmentation

The nuclei in the whole slide image were automatically segmented using Tissue Gnostics analysis software. We determined the optimal module parameters for nuclei detection, such as different dye weight [4′,6-diamidino-2-phenylindole (DAPI) nuclear weight = 5, opal 520 nucleus weight = 0. 15, opal 570 nucleus weight = 0.243, opal 690 nucleus weight = 0.113, nuclear contrast threshold = 0.4, minimum nuclear intensity = 0.032, nuclear segmentation aggressiveness = 0.75, and default nuclear size setting = 1-450 μm^2^].

### Recognition and analysis of tumor-infiltrating CD8^+^ T lymphocytes, CD133^+^ CSCs, and CK19^+^ TB

After segmenting the nuclei (DAPI) under the same analysis module, the cells were then classified as CD8-positivity (opal 520) based on the dye nucleus- (opal 520 0.344), cytoplasm- (opal 520 0.025), and membrane-positive thresholds (opal 520 0.025). CD133-positivity (opal 570) was based on dye nucleus- (opal 570 0.524), cytoplasm- (opal 570 0.045), and membrane-positive thresholds (opal 570 0.045). CK19-positivity (opal 690) was based on dye nucleus- (opal 690 0.624), cytoplasm- (opal 690 0.025), and membrane-positive thresholds (opal 690 0.025). CK19^+^ TB and CK19^+^ PDAC ducts were recognized based on the number of nuclei and morphology. Consistent settings of the analysis algorithm were applied to all slides prepared from patient specimens. The algorithm was used to automatically quantify the number of each lymphocyte classification across the entire slide image.

### Spatial analysis of TB, CSCs, and CD8^+^ T cells

The spatial distributions of TB, CSCs, and CD8^+^ T cells were imported into a spatial dot plot within the Tissue Gnostics software. According to this plot, the spatial analysis algorithm was utilized to calculate the number of CD8^+^ T cells between 0 and 100 μm radii of TB or CSCs in consecutive increments of 20 μm, thus creating 5 classes, including 0–20, 20–40, 40–60, 60–80, and 80–100 μm. Finally, the number and densities of CD8^+^ T cells within these distances were established.

### Flow cytometry

Fresh PDAC specimens and *in vivo* humanized patient-derived xenograft (PDX) specimens were collected and immediately digested into single cell suspensions with 1 mg/mL of collagenase (C2799; Sigma-Aldrich, City of Saint Louis, State of Missouri, USA), 2.5 U/mL of hyaluronidase (H3506; Sigma-Aldrich), and 0.1 mg/mL of DNase Ⅰ (DN25; Sigma-Aldrich). The single cell suspensions were stained with anti-CD8 (300922; Biolegend, City of Santiago, State of California, USA) and anti-CD133 antibodies (372806; Biolegend). Isotype controls were used as negative controls. The data were analyzed using soft Flow Jo 10.0 (BD Biosciences, City of Franklin Lakes, State of New Jersey, USA).

### Statistical analysis

Statistical analyses were performed using IBM SPSS Statistics (version 21.0; City of Chicago, State of Illinois, USA). The cut-off values for each index were determined according to the median values in the PDAC patient cohorts. Categorical variables were compared using a *χ*^2^ test, while continuous variables were compared using a *t*-test. The correlation between IHC scoring and IF counting results was estimated using Pearson coefficient analysis. Survival curves were estimated using the Kaplan–Meier method and compared using a log-rank test. Univariate and multivariate Cox analyses were performed to determine the independent risk characteristics. The hazard ratios (HRs) and 95% confidence intervals (CIs) of these variables were estimated to quantify the strength of the associations. The nomogram, calibration curve, time-dependent receiver operating characteristic (ROC) curve, and decision curve analyses were performed with R software (University of Auckland, City of Auckland, New Zealand). All statistical tests were 2-tailed. Statistical significance was considered at a *P* < 0.05.

## Results

### AI-based automatic recognition of tumor-infiltrating CD8^+^ T cells, CD133^+^ CSCs, and TB in patients with PDAC

**Figure 1:**
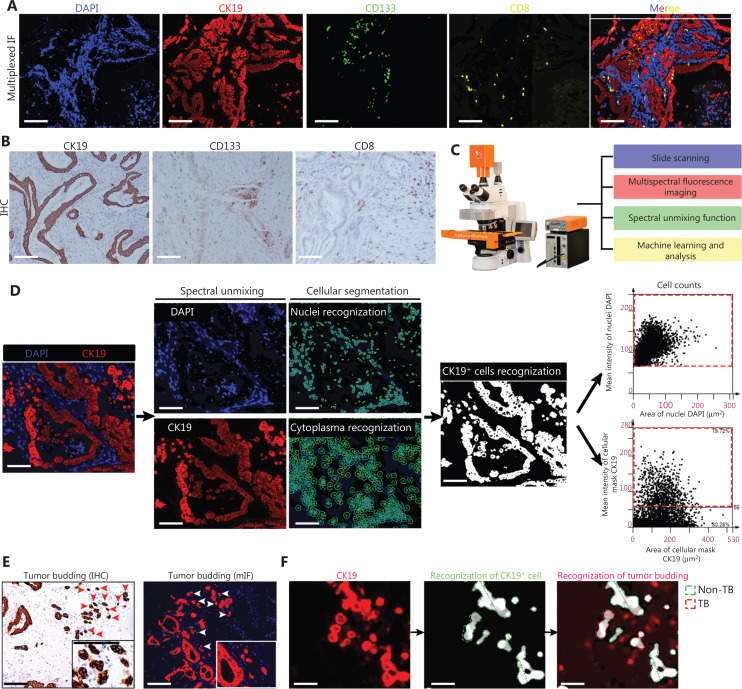

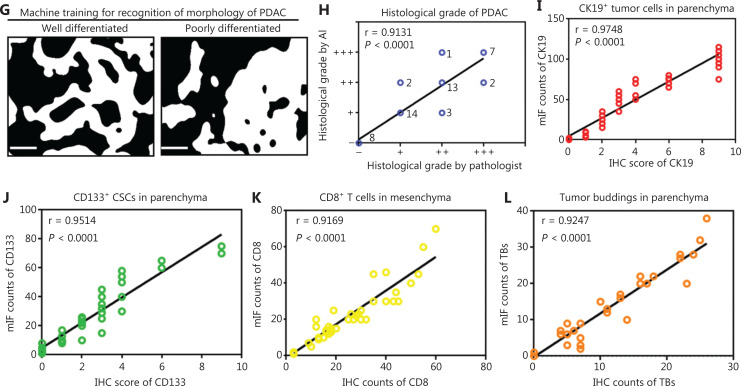
Automatic recognition of tumor-infiltrating CD8^+^ T cells, CD133^+^ CSCs, and CK19^+^ tumor budding in PDAC based on artificial intelligence. Images in Panels A, D, E, F, and G were all obtained from the same slide of the same PDAC patient and used as an example to illustrate the analytic process. (A) Representative mIF images of PDAC tissues. CK19^+^ tumor cells, CD133^+^ CSCs, CD8^+^ T cells, and nuclei are annotated in red, green, yellow, and blue, respectively. Scale bar, 100 μm. (B) Representative IHC images of PDAC tissues for CK19, CD133, and CD8. Scale bar, 100 μm. (C) Brief introduction of whole landscape quantitative analysis system of Tissue Gnostics (Zeiss). (D) Schematic illustration for cellular segmentation, CK19^+^ cell recognition, and CK19^+^ cell counting based on an automatic system. The DAPI^+^ and CK19^+^ cells are presented as dot plots. The red rectangle frame (formed by dash lines) in the upper dot plots represents DAPI-positive cell subsets based on the threshold of DAPI intensity and the red rectangle frame (formed by dash lines) in the lower dot plots represents CK19-positive cells subsets based on the threshold of CK19 intensity. Scale bar, 100 μm. (E) Representative IHC and mIF images for CK19^+^ tumor budding detection. The red arrows indicate CK19^+^ tumor budding determined by CK19 IHC staining and the white arrows indicate CK19^+^ tumor budding determined by CK19 mIF staining. Scale bar, 100 μm. (F) Recognition of CK19^+^ tumor budding based on automatic system. The region formed by green dash lines indicate CK19^+^ tumor cells and the region formed by red dash lines indicate tumor budding. Scale bar, 100 μm. (G, H) Recognition of PDAC with different histologic grades based on machine learning. The CK19^+^ PDAC was recognized and marked with a white mask by artificial intelligence. Consistency between results of IHC evaluation by experienced pathologists and number of mIF assessments by machine learning was compared and a Spearman correlation test was performed (H). The number next to the blue points in Figure 2H mean patient sample counts with the indicated IHC grade. (I–L) Consistency between results of the IHC evaluation by experienced pathologists and the number of mIF assessments by automatic counting was compared and the Spearman correlation test was performed. CK19^+^ tumor cells (I), CD133^+^ CSCs (J), CD8^+^ T cells (K), and tumor budding (L).

Based on the pivotal roles of CD8^+^ T cells and CD133^+^ CSCs on the TME, the infiltrated CD8^+^ T cells and CD133^+^ CSCs were assessed using multi-color immunofluorescence (IF) and IHC staining (**Figure 1A and 1B**). The expression of CK19 indicated tumor parenchyma in the multi-color IF analysis. AI analysis via the whole landscape quantitative analysis system of Tissue Gnostics was performed (**Figure 1C**). A detailed description of the AI-based system is illustrated as a workflow and shown in **Supplementary Figure S1A**. As shown in **Figure 1D**, quantifying CK19^+^ cancer cells within the tumor regions chosen as an example, nuclei were detected using this system and cancer cells were classified based on CK19-positivity in the nucleus, cytoplasm, and membrane. The number of CK19^+^ cancer cells was presented as dot plots according to the IF density threshold in each segmentation. The cancer cell classification thresholds were kept constant across the entire analysis of the cohort. The CD8^+^ T cells and CD133^+^ CSCs were also recognized based on the fluorescence intensities of CD8 and CD133 proteins. Importantly, a TB is typically defined as a single tumor cell or tumor cell cluster of up to five cells at the invasive front. Thus, we defined CK19^+^ single cells or CK19^+^ clusters of whole nuclei (*n* = 1-5) as a TB (**Figure 1E**). The CK19^+^ TB was successfully recognized by AI based on the number of cluster nuclei (**Figure 1F**). We also trained the Tissue Gnostics system to recognize PDAC with a typical well-differentiated regular duct structure or poorly differentiated morphology (**Figure 1G**). Spearman correlation coefficient analysis was performed to compare agreement of the results between AI and experienced pathologists. Good agreement was acquired between experienced pathologists and automatic recognition by software (**Figure 1H**). The consistency between the IHC score evaluation results by experienced pathologists and the number of multiplex immunofluorescence (mIF) counting by software was compared, and the results showed good agreement between the two approaches (**Figure 1I–1L**). Furthermore, to evaluate the diagnostic performance of the AI-based recognition system, the specificity, sensitivity, and Youden indices were all analyzed for the AI-based recognition system. The results recognized by experienced pathologists were determined as “true positive” and “true negative.” As shown in **Supplementary Figure S1B–S1D**, the AI-based recognition system showed high level of specificity, sensitivity, and Youden indices, indicating good diagnostic performance of the AI-based recognition system for CD133^+^ CSCs, TB, and CD8^+^ T cells.

### Correlation of tumor-infiltrating CD8^+^ T cells, CD133^+^ CSCs and TBs and clinical outcome

**Figure 2:**
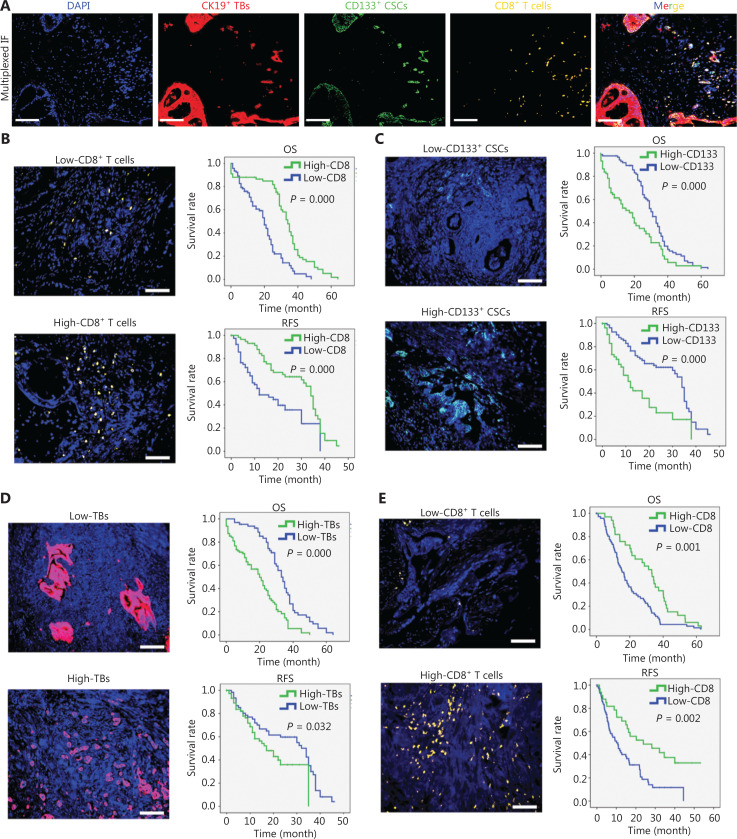

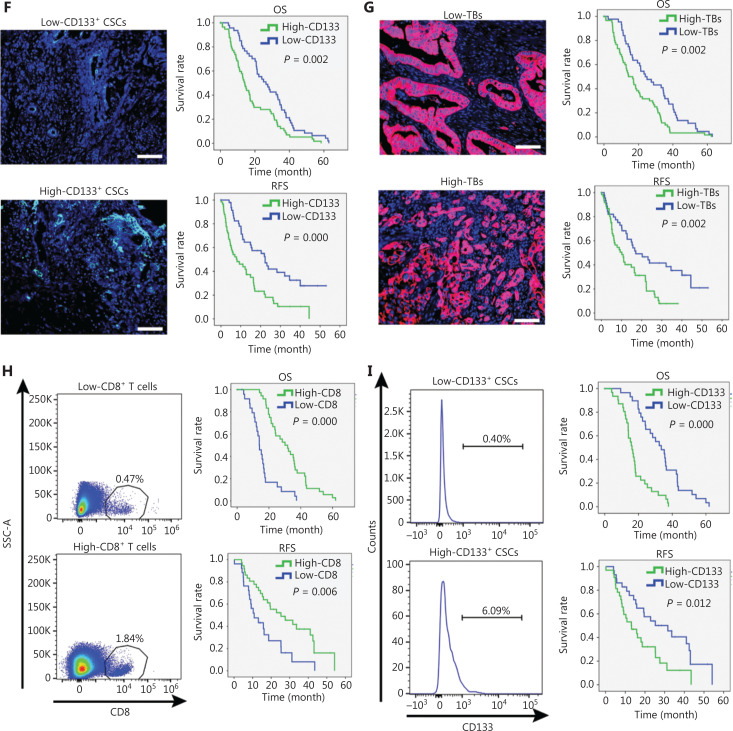

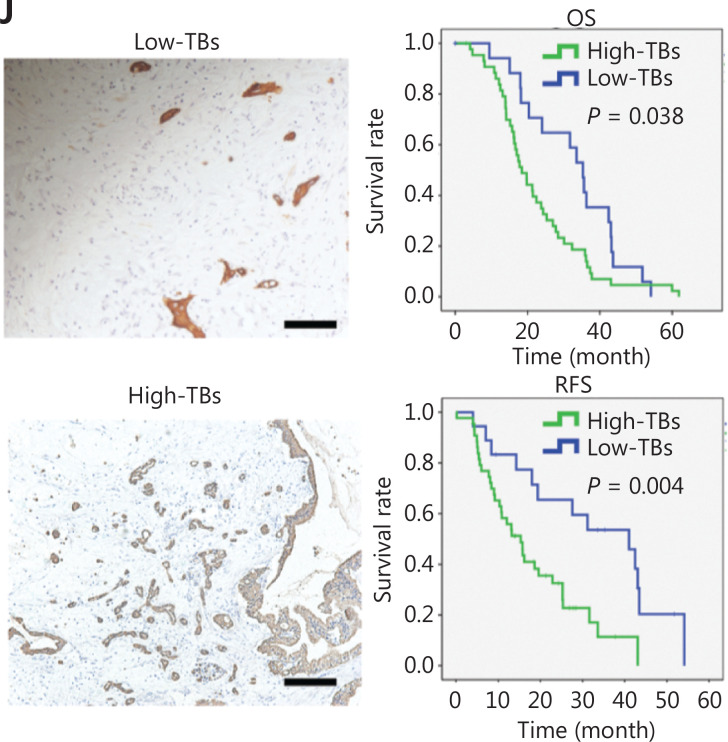
Correlation of tumor-infiltrating CD8^+^ T cells, CD133^+^ CSCs, and tumor budding, and clinical outcome. (A) Representative mIF images for co-staining of CK19, CD8, and CD133 on the same slide. CD8 in yellow, CK19 in red, CD133 in green, and nuclei in blue for multi-color IF staining. Scale bar, 100 μm. (B) mIF staining for low and high CD8^+^ T cells in a retrospective primary training cohort (left). Scale bar, 100 μm. CD8 in yellow and nuclei in blue for multi-color IF staining. Overall survival and relapse-free survival of patients grouped by different CD8^+^ T cell infiltrating status (right). (C) mIF staining for CD133^+^ CSCs in a retrospective primary training cohort (left). CD133 in green and nuclei in blue for multi-color IF staining. Scale bar, 100 μm. Overall survival and relapse-free survival of patients grouped by different CD133^+^ CSC infiltrating status (right). (D) mIF staining for CK19^+^ tumor budding in a retrospective primary training cohort (left). CK19 in red and nuclei in blue for multi-color IF staining. Scale bar, 100 μm. Overall survival and relapse-free survival of patients grouped by different CK19^+^ tumor budding infiltrating status (right). (E) mIF staining for CD8^+^ T cells in a retrospective validation cohort (left). CD8 in yellow and nuclei in blue for multi-color IF staining. Scale bar, 100 μm. Overall survival and relapse-free survival of patients grouped by different CD8^+^ T cell infiltrating status (right). (F) mIF staining for CD133^+^ CSCs in a retrospective validation cohort (left). CD133 in green and nuclei in blue for multi-color IF staining. Scale bar, 100 μm. Overall survival and relapse-free survival of patients grouped by different CD133^+^ CSC infiltrating status (right). (G) mIF staining for CK19^+^ tumor budding in a retrospective validation cohort (left). CK19 in red and nuclei in blue for multi-color IF staining. Scale bar, 100 μm. Overall survival and relapse-free survival of patients grouped by different CK19^+^ tumor budding infiltrating status (right). (H) Flow cytometry detection for CD8^+^ T cells of fresh specimens from another prospective validation cohort. Representative dot plots are shown (left). Gated at CD45^+^ cells. Overall survival and relapse-free survival of patients grouped by different CD8^+^ T cell infiltrating status (right). (I) Flow cytometry detection for CD133^+^ CSCs of fresh specimens from another prospective validation cohort. Representative histograms are shown (left). Gated at EpCAM^+^ epithelial cells. Overall survival and relapse-free survival of patients grouped by different CD133^+^ CSC infiltrating status (right). (J) IHC staining for CK19^+^ tumor budding in another prospective validation cohort. Representative IHC image of tumor budding are shown (left). Scale bar, 100 μm. Overall survival and relapse-free survival of patients grouped by different CK19^+^ tumor budding infiltrating status (right).

PDAC specimens from the primary cohort were stained with multi-color IF staining for CD8, CD133, and CK19 to determine the significance of tumor-infiltrating CD8^+^ T cells, CD133^+^ CSCs, and TB with respect to survival of patients with PDAC (**Figure 2A**). Statistical analysis based on the AI Tissue Gnostics system was subsequently performed. Tissue samples from patients with PDAC in the primary training cohort were divided into two groups based on the density of CD8^+^ T cells, CD133^+^ CSCs, or CK19^+^ TB as follows: a low-density CD8^+^ T cell group (CD8^+^ T cell count/mm^2^ ≤ 309.2) and a high-density CD8^+^ T cell group (CD8^+^ T cell count/mm^2^ > 309.2); a low-density CD133^+^ T cell group (CD133^+^ cell count/mm^2^ ≤ 131.6) and a high-density CD133^+^ cell group (CD133^+^ cell count/mm^2^ > 131.6); and a low-density TB group (TB count/mm^2^ ≤ 336.1) and a high-density TB group (TB count/mm^2^ > 336.1). Representative multi-color IF staining images of PDAC from the primary training cohort are shown in **Figure 2B**. The median overall survival (OS) and relapse-free survival (RFS) in the low CD8^+^ T cell index group were significantly shorter than the high CD8^+^ T cell index group. In addition, the PDAC patients with high densities of CD133^+^ CSCs and TB had significantly poorer OS and RFS than patients with low densities of CD133^+^ CSCs and TB (**Figure 2C and 2D**). Consistent results were obtained with another retrospective validation cohort (**Figure 2E–2G**). A *χ*^2^ test and Spearman correlation test were performed to determine the association between the densities of CD8^+^ T cell, CD133^+^ cells, and TB with clinical and pathologic parameters in PDAC. As shown in **Supplementary Table S4**, the density of CD8^+^ T cells, CD133^+^ cells, and TB had no correlation with age, gender, and nerve invasion. Notably, the density of CD8^+^ T cells was negatively correlated with tumor size, histologic grade, TNM stage, lymph node metastases, and CA19-9 concentration, whereas the densities of CD133^+^ CSCs and TB were positively correlated with tumor size, histologic grade, TNM stage, lymph node metastases, and CA19-9 concentration. Furthermore, univariate and multivariate analyses suggested that the densities of CD8^+^ T cells, CD133^+^ CSCs, and TB were independent prognostic factors in patients with PDAC (**Supplementary Table S5**). These results were in agreement with the anti-tumor activity of cytotoxic CD8^+^ T cells and the pro-tumor activities of CD133^+^ CSCs and TB. Another validation cohort of patients with PDAC was used to confirm the analysis results; consistent results were obtained (**Supplementary Tables S6 and S7)**. We also collected 63 fresh PDAC specimens from another prospective validation cohort, analyzed the tumor-infiltrating CD8^+^ T cells and CD133^+^ CSCs by flow cytometry and detected TB using IHC staining. Consistent results were obtained from a prospective cohort study using fresh specimens from patients with PDAC (**Figure 2H–2J**).

Additionally, consistent results were observed from a different independent validated PDAC cohort from another center (Department of Hepatopancreatobiliary Surgery, Tongliao City Hospital; **Supplementary Figure S2A and S2B**).

### Relationship between the densities of tumor-infiltrating CD8^+^ T cells, CD133^+^ CSCs, and TB

Immune evasion, CSCs, and TB all contributed to tumor growth and metastasis^20,21,26,27^. A high-stemness signature correlates with a poor immunogenic response in some solid malignancies, highlighting the potential interplay between CSCs and tumor immune cells^28^. Zlobec et al.^29^ reported that the absence of tumor-infiltrating lymphocytes (TILs) is highly correlated with the presence of TB, indicating that TB may restrict an anti-tumor host immune response and further contribute to immune evasion. Thus, we next evaluated the relationship among the densities of CD8^+^ T lymphocytes, CD133^+^ CSCs, and TB using a *χ*^2^ test and Spearman correlation coefficient analysis. As shown in **Supplementary Figure S3A–S3C**, a negative relationship was observed between the densities of CD8^+^ T lymphocytes and TB or CD133^+^ CSCs in the primary retrospective training cohort, whereas a positive relationship existed between the densities of TB and CD133^+^ CSCs. Consistent results were also obtained with the retrospective validation cohort (**Supplementary Figure S3D–S3F**). We collected fresh PDAC specimens post-operatively, evaluated the tumor-infiltrating CD8^+^ T cells, and CD133^+^ CSCs via flow cytometry and assessed TB using IHC. As shown in **Supplementary Figure S3G–S3I**, the percentage of CD8^+^ T cells was inversely correlated with the percentages of CD133^+^ CSCs and TB, whereas the percentage of CD133^+^ CSCs was positively correlated with the percentage of TB. Moreover, we determined the correlation between exhausted CD8^+^ T cells and CSCs/TB in patients with PDAC. As shown in **Supplementary Figure S3J–S3Q**, the proportions of PD-1^+^CD8^+^ T cells and Tim-3^+^CD8^+^ T cells were positively correlated with CD133^+^ CSCs and CK19^+^ TB in the retrospective primary training and validation cohorts from our center.

### Correlations between tumor-infiltrating CD8^+^ T cell/sTB index, CD8^+^ T cells/CD133^+^ CSC index, and clinical outcome

**Figure 3:**
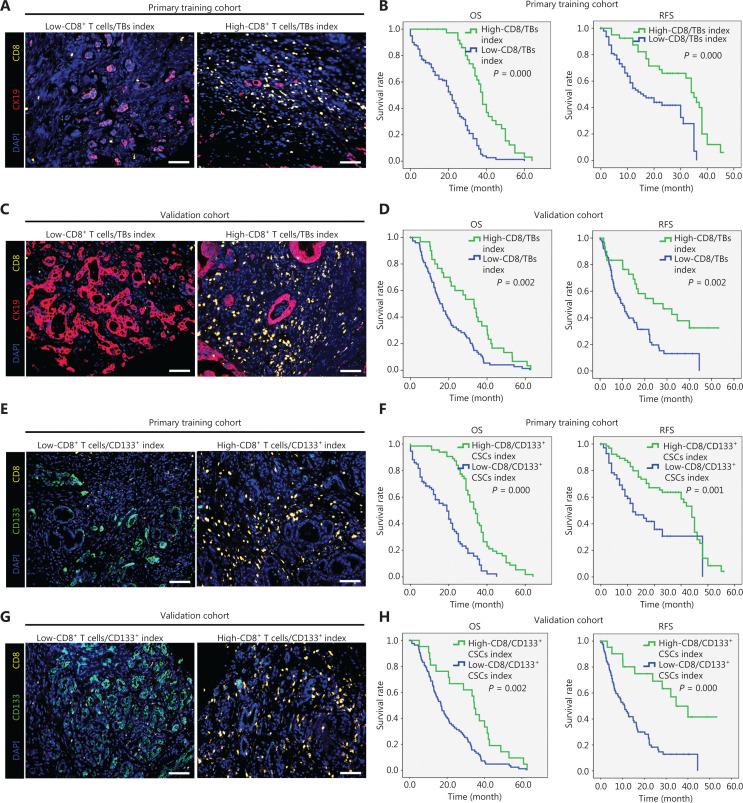
Correlation of tumor-infiltrating CD8^+^ T cell/tumor budding and CD8^+^ T cell/CD133^+^ CSC indices and clinical outcome. (A, B) Representative mIF staining for CD8^+^ T cells and CK19^+^ tumor budding in a retrospective primary training cohort (A). CD8 in yellow, CK19 in red, and nuclei in blue. Scale bar, 100 μm. Overall survival and relapse-free survival of patients grouped by different CD8^+^ T cell/CK19^+^ tumor budding index (B). (C, D) Representative mIF staining for CD8^+^ T cells and CK19^+^ tumor budding in a retrospective validation cohort (C). CD8 in yellow, CK19 in red, and nuclei in blue. Scale bar, 100 μm. Overall survival and relapse-free survival of patients grouped by different CD8^+^ T cell/CK19^+^ tumor budding indices (D). (E, F) Representative mIF staining for CD8^+^ T cells and CD133^+^ CSCs in a retrospective primary training cohort (E). CD8 in yellow, CD133 in green, and nuclei in blue. Scale bar, 100 μm. Overall survival and relapse-free survival of patients grouped by different CD8^+^ T cell/CD133^+^ CSC indices (F). (G, H) Representative mIF staining for CD8^+^ T cells and CD133^+^ CSCs in a retrospective validation cohort (G). CD8 in yellow, CD133 in green, and nuclei in blue. Scale bar, 100 μm. Overall survival and relapse-free survival of patients grouped by different CD8^+^ T cell/CD133^+^ CSC indices (H).

Lugli et al.^30^ reported that the CD8^+^ T cells/TB index can be used as an independent prognostic factor representing an ‘attacker-defender’ model in the TME of patients with colorectal cancer (CRC); however, to date most investigations involving TB and CD8^+^ T cell infiltrating PDAC have been conducted independently. Thus, we established an ‘anti-/pro-tumor’ model representing the tumor-infiltrating CD8^+^ T cell/TB index in patients with PDAC. Tissue samples from patients with PDAC in the primary training cohort were divided into two groups according to the CD8^+^ T cell/TB index as follows: a low CD8^+^ T cell/TB index group (index ≤ 3.73); and a high CD8^+^ T cell/TB index group (index > 3.73). As shown in **Figure 3A and 3B**, the median OS and RFS in the low CD8^+^ T cell/TB index group was significantly shorter than the high CD8^+^ T cell/TB index group. Consistent results were observed in another validation cohort (**Figure 3C and 3D**). Recently, the cross-talk between CSCs and immune cells has provided a paradigm for the reciprocal interaction between different components in the TME. Thus, we also established another ‘anti-/pro-tumor model’ represented by tumor-infiltrating CD8^+^ T cells/CD133^+^ CSCs in patients with PDAC. Tissue samples from patients with PDAC in the primary training cohort were divided into two groups according to the CD8^+^ T cell/CD133^+^ CSC index, as follows: a low CD8^+^ T cell/CD133^+^ CSCs index group (index ≤ 3.325); and a high CD8^+^ T cell/CD133^+^ CSC index group (index > 3.325). As shown in **Figure 3E and 3F**, patients with PDAC and a low CD8^+^ T cell/CD133^+^ CSC index had significantly poorer OS and RFS than patients with high CD8^+^ T cell/CD133^+^ CSC indices. Consistent results were observed in another validation cohort (**Figure 3G and 3H**). Furthermore, a *χ*^2^ test and Spearman correlation test were performed to determine the association between the CD8^+^ T cell/TB index, CD8^+^ T cell/CD133^+^ CSC index, and clinicopathologic parameters in PDAC. As shown in **Supplementary Table S4**, the CD8^+^ T cell/TB and CD8^+^ T cell/CD133^+^ CSC indices had no correlation with age, gender, and nerve invasion. Importantly, the two indices were negatively correlated with tumor size, TNM stage, lymph node metastases, and CA19-9 concentration, while being positively correlated with histologic grade. Moreover, univariate and multivariate analyses suggested that the CD8^+^ T cell/TB and CD8^+^ T cell/CD133^+^ CSC indices were independent prognostic factors in PDAC (**Supplementary Table S5**). Consistent results were also observed in another retrospective validation cohort (**Supplementary Tables S6 and S7**).

Additionally, consistent results were also observed from a different independent PDAC cohort from another center (Department of Hepatopancreatobiliary Surgery, Tongliao City Hospital; **Supplementary Figure S2A and S2B**).

### Correlations between TB-adjacent CD8^+^ T cells and CD133^+^ CSC-adjacent CD8^+^ T cells, and clinical outcome

**Figure 4:**
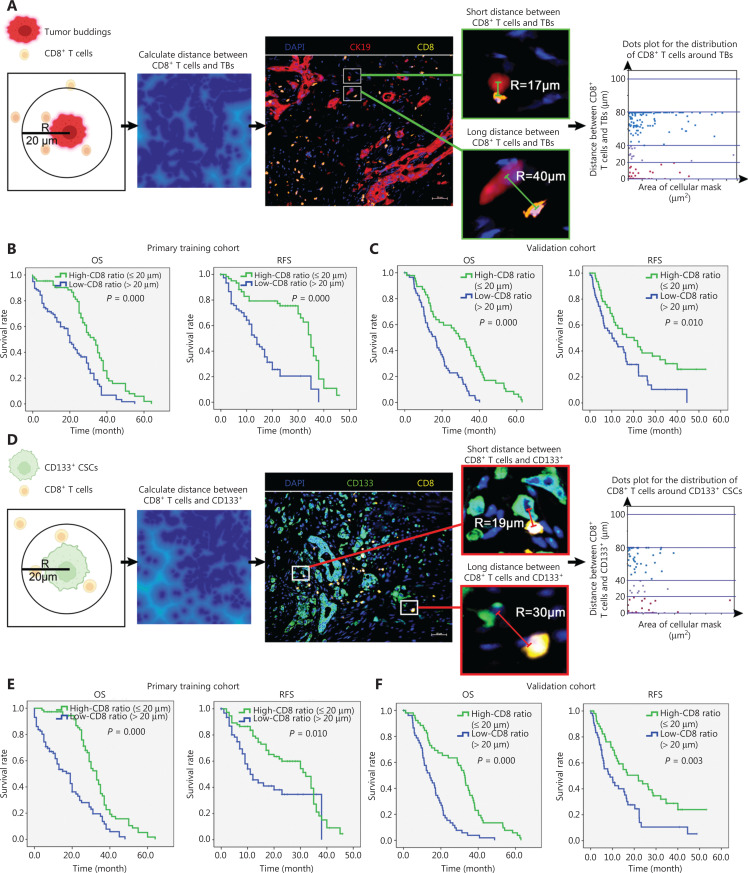
Correlation of tumor budding-adjacent CD8^+^ T cell and CD133^+^ CSC-adjacent CD8^+^ T cell ratios and clinical outcome. (A) Schematic illustration for recognition and percentage analysis of tumor budding-adjacent CD8^+^ T cells within a radius of 20 μm from the nuclear center of CK19^+^ tumor budding based on our automatic machine counting. The distance between CD8^+^ T cells and tumor budding was calculated, then the distribution of CD8^+^ T cells within a radius of 20, 40, 80. and 100 μm is shown in a dot plot. (B) Overall survival and relapse-free survival of patients grouped according to the tumor budding-adjacent CD8^+^ T cell ratio in a retrospective primary training cohort. (C) Overall survival and relapse-free survival of patients grouped according to the tumor budding-adjacent CD8^+^ T cell ratio in a retrospective validation cohort. (D) Schematic illustration for recognition and percentage analysis of CD133^+^ CSC-adjacent CD8^+^ T cells within a radius of 20 μm from the nuclear center of CD133^+^ CSCs based on our automatic machine counting. The distance between CD8^+^ T cells and CD133^+^ CSCs was calculated, then the distribution of CD8^+^ T cells within a radius of 20, 40, 80, and 100 μm is shown in a dot plot. Representative CD133^+^ CSCs-adjacent CD8^+^ T cells (R = 19 μm) and CD133^+^ CSCs-distal CD8^+^ T cells (R = 30 μm) are shown. (E) Overall survival and relapse-free survival of patients according to the CD133^+^ CSC-adjacent CD8^+^ T cell ratio in a retrospective primary training cohort. (F) Overall survival and relapse-free survival of patients according to the CD133^+^ CSC-adjacent CD8^+^ T cell ratio in a retrospective validation cohort.

Carstens et al.^16^ reported that the spatial distribution of intra-tumoral CD8^+^ T cells correlates with tumor progression and prognosis of patients with pancreatic cancer. Thus, we hypothesized that the spatial distribution of intra-tumoral CD8^+^ T cells with respect to TB or CD133^+^ CSCs correlated with patient outcome. Based on our hypothesis, we used Ripley’s L-function model to evaluate the distribution of CD8^+^ T cells relative to CK19^+^ TB or CD133^+^ CSCs using AI analysis (**Figure 4A and 4D**). An L-function is defined, in this case, as the number of CD8^+^ T cells within a specified radius (*r*) distributed from a given point (nuclear center of CK19^+^ TB cells or CD133^+^ CSCs) and is represented as follows: π*L(r_0_)^2^. If the T-cell population is randomly distributed relative to the CK19^+^ TB cells and CD133^+^ CSCs, the underlying theoretical L-function will have the form, L(r_0_) = r, represented by a linear slope. The spatial distribution of the CK19^+^ TB and CD133^+^ CSCs was imported into a spatial plot within the Tissue Gnostics software. From this plot, the spatial analysis algorithm was utilized to calculate the amount of TB between 0 and 100 μm radii of a PDAC (**Figure 4A and 4D**) in consecutive increments of 20 μm, thereby creating 5 classes: 0–20; 20–40; 40–60; 60–80; and 80–100 μm. We focused on a 20-μm radius around CK19^+^ TB or CD133^+^ CSCs, representing an increased probability of direct cell–cell contact (**Figure 4A and 4D**). The ratio of CD8^+^ T cells within the above radius size was calculated and the prognosis significance was analyzed. Tissue samples from patients with PDAC in the primary training cohort were first divided into two groups according to the TB-adjacent CD8^+^ T cell ratio within 20 μm relative to the CK19^+^ TB cell center, as follows: a low CD8^+^ T cell ratio group (index ≤ 0.21); and a high CD8^+^ T cell ratio group (index > 0.21). As shown in **Figure 4B**, the median OS and RFS in the high CD8^+^ T cell ratio group were significantly longer than the low CD8^+^ T cell ratio group. These findings were in agreement with the required cell-cell contact necessary for cytotoxic CD8^+^ T cell anti-tumor activity. Consistent results were observed in another retrospective validation cohort (**Figure 4C**). Moreover, we divided the primary training cohort into two groups based on the CD133^+^ CSC-adjacent CD8^+^ T cell ratio within 20 μm relative to the CD133^+^ CSC center, as follows: a low CD8^+^ T cell ratio group (index ≤ 0.19); and a high CD8^+^ T cell ratio group (index > 0.19). As shown in **Figure 4E**, the median OS and RFS in the low CD8^+^ T cell ratio group were shorter than the high CD8^+^ T cell ratio group. Consistent results were also observed in another retrospective validation cohort (**Figure 4F**).

Additionally, the correlation between TB-adjacent CD8^+^ T cells and CD133^+^ CSC-adjacent CD8^+^ T cells in PDAC was determined. As shown in **Supplementary Figure S4A and S4B**, a significantly positive correlation was observed between the two variables in the retrospective primary and validation cohorts. Considering the positive correlation between CD133^+^ CSCs and CK19^+^ TB in patients with PDAC (**Supplementary Figure S3C, S3F, & S3I**), we concluded that CD133^+^ CSCs have a high metastatic potential. In addition, we also observed that partial CD133^+^ CSCs were co-localized with CK19^+^ TB based on mIF staining (**Supplementary Figure S4C**), which further indicated the partial overlap between TB-adjacent CD8^+^ T cells and CD133^+^ CSC-adjacent CD8^+^ T cells. Moreover, we also determined the spatial localization of exhausted CD8^+^ T cells surrounding CSCs and TB. As shown in **Supplementary Figure S5A–S5H**, most exhausted PD1^+^CD8^+^ T cells and Tim-3^+^CD8^+^ T cells were distributed within 20–40 μm relative to the nuclear center of CK19^+^TB and CD133^+^ CSCs in the retrospective primary training cohort.

Additionally, consistent results were observed from a different independent validated PDAC cohort from another center (Department of Hepatopancreatobiliary Surgery, Tongliao City Hospital; **Supplementary Figure S2A and S2B**).

### *In vivo* validation of the associations between CD8^+^ T lymphocytes, CD133^+^ CSCs, and TB in a humanized mouse model

**Figure 5:**
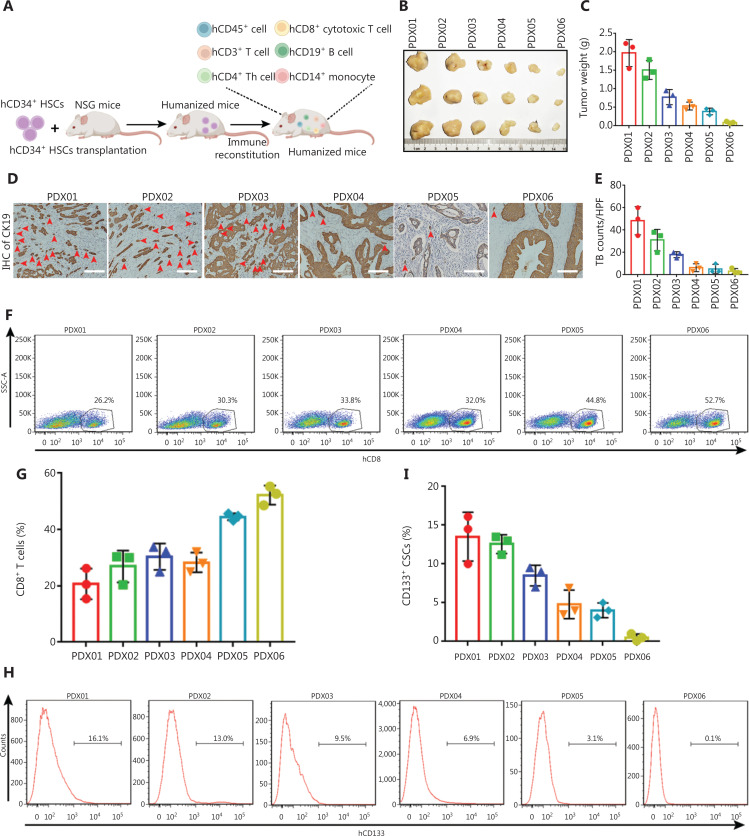

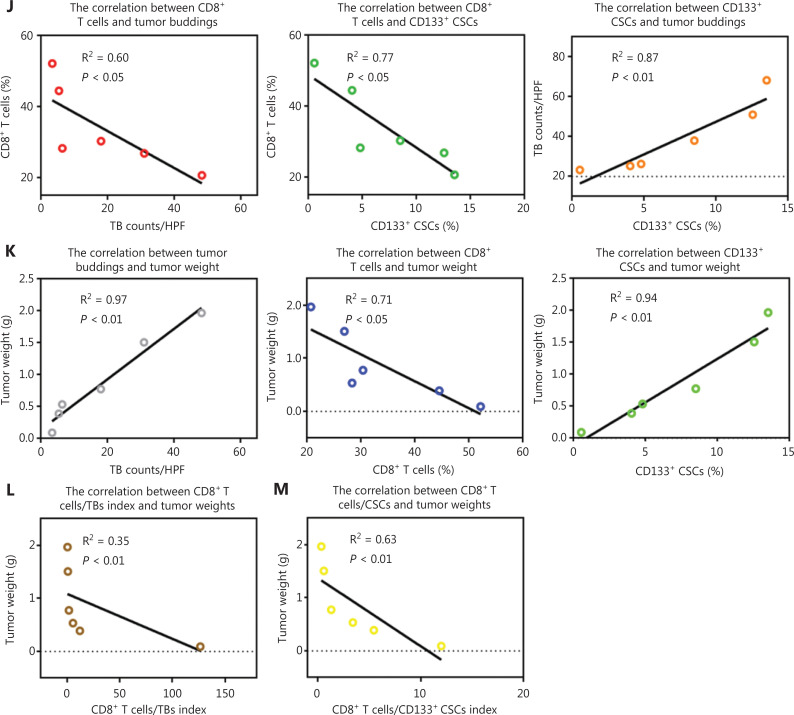
*In vivo* validation for the association between CD8^+^ T lymphocytes, CD133^+^ CSCs, and tumor budding in a humanized mouse model. (A) The schematic illustration for the establishment of a humanized mouse model. (B, C) Patient-derived xenografts (PDXs) from six patients with PDAC were subcutaneously transplanted into a humanized mouse model. Mice were sacrificed after 2 months. Representative tumor images (B) and tumor weights (C) are shown. (D, E) Tumor budding in PDXs was determined by CK19 IHC staining. Images of representative tumor budding images of PDXs are shown (D) and the density of tumor budding per high power field was analyzed (E). Red arrows indicated CK19^+^ tumor budding. Scale bar, 100 μm. (F, G) Fresh PDX tumors were digested into single cell suspensions, and tumor-infiltrating human CD8^+^ T cells were determined by flow cytometry. Representative dot plots of human CD8^+^ T lymphocytes are shown (F), and the percentage of tumor-infiltrating human CD8^+^ T cells were analyzed (G). Gated at human CD45^+^ cells. (H, I) Fresh PDX tumors were digested into single cell suspensions and tumor-infiltrating human CD133^+^ CSCs were determined by flow cytometry. Representative dot plots of human CD133^+^ CSCs are shown (H), and the percentage of tumor-infiltrating human CD133^+^ CSCs were analyzed (I). Gated at human EpCAM^+^ epithelial cells. (J) Correlation between the percentage of tumor-infiltrating human CD8^+^ T cells, human CD133^+^ CSCs, and density of tumor budding. Spearman correlation analysis was performed. (K) Correlation between the percentage of tumor-infiltrating human CD8^+^ T cells, human CD133^+^ CSCs, density of tumor budding, and PDX tumor weights. Spearman correlation analysis was performed. (L, M) mIF staining for human CD8, human CD133, and human CK19 was performed on all PDX tumor slides, then all mIF slides of PDXs were automatically analyzed using the Tissue Gnostic system. Correlation between tumor-infiltrating human CD8^+^ T cells/tumor budding index (L), tumor-infiltrating human CD8^+^ T cell/CD133^+^ CSC index (M), and PDX tumor weights. Spearman correlation analysis was performed.

To further validate our findings, we used human PDXs from six individual patients to evaluate the associations between TB, human CD133^+^ CSCs, and human CD8^+^ T lymphocytes. To mimic the human immune system, we utilized a humanized mouse model established by reconstituting the immune system from human CD34^+^ hemopoietic stem cells (HSCs) and subcutaneously transplanting PDXs (**Figure 5A**). After 2 months, all mice were sacrificed and representative tumor images and tumor weights were determined (**Figure 5B and 5C**). Flow cytometry was performed to quantity the tumor-infiltrating human CD8^+^ lymphocytes and tumor-infiltrating human CD133^+^ CSCs. As shown in **Figure 5D and 5E**, PDX01, PDX02, and PDX03 exhibited high densities of TB, while PDX04, PDX05, and PDX06 showed low densities of TB. As expected, PDX01, PDX02, and PDX03 exhibited low percentages of tumor-infiltrating human CD8^+^ T lymphocytes and high percentages of tumor-infiltrating human CD133^+^ CSCs, whereas PDX04, PDX05, and PDX06 exhibited high percentages of tumor-infiltrating human CD8^+^ T lymphocytes and low percentages of tumor-infiltrating human CD133^+^ CSCs (**Figure 5F–5I**). Spearman correlation coefficient analysis of the PDX results further validated the strongly negative correlation between CD8^+^ T cells and TB, the negative correlation between CD133^+^ CSCs and CD8^+^ T cells, and the positive correlation between CD133^+^ CSCs and TB (**Figure 5J**). Importantly, the tumor weights of the PDXs were positively correlated with the percentages of CD133 CSCs and TB, and negatively correlated with the percentage of CD8^+^ T cells (**Figure 5K**). We also evaluated CD8^+^ T cells, CD133^+^ CSCs, and CK19^+^ TB on the same tissue section via AI-based automated analysis on the Tissue Gnostics system. PDX mice with low CD8^+^ T cell/CD133^+^ CSC and CD8^+^ T cell/TB indices had a larger tumor burden than PDX mice with high values of these indices (**Figure 5L and 5M**).

### Development of a nomogram-based multi-parameter profile and validation

**Figure 6:**
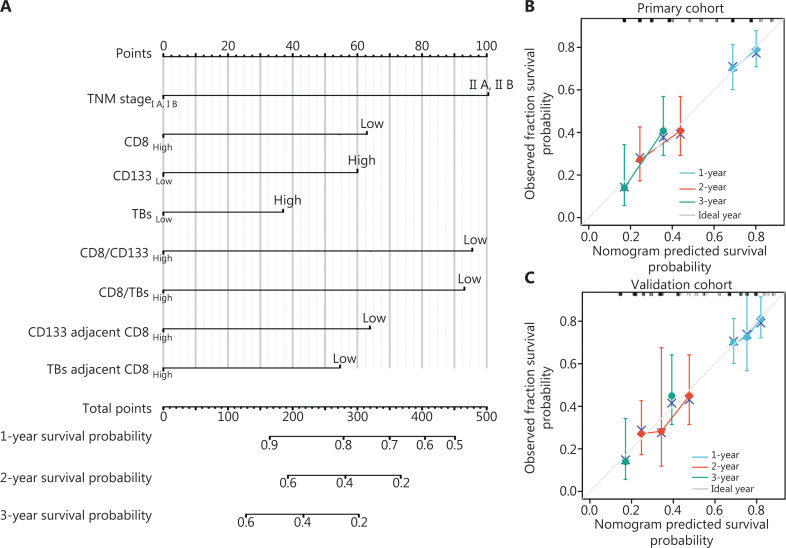
Development of nomogram-based multi-parameter profile and validation. (A) Nomogram for predicting overall survival in patients with PDAC after surgery. To estimate the survival rate of an individual patient, the value of each factor was acquired on each variable axis, followed by a line drawn straight upward to determine the points. The sum of these eight numbers is located on the Total Points axis, then a line is drawn downward to the survival axis to determine the likelihood of survival. (B, C) Calibration curves for predicting the overall survival (OS) of patients in a retrospective primary cohort (B) and retrospective validation cohort (C). Nomogram-predicted probability of survival is plotted on the x-axis, and actual survival is plotted on the y-axis.

Although all eight parameters for TNM stage, immune, CSCs, and TB, including TNM stage, CD8^+^ T cells, CD133^+^ CSCs, TB, CD8^+^ T cell/CD133^+^ CSC, CD8^+^ T cell/TB, TB-adjacent CD8^+^ T cell, and CD133^+^ CSC-adjacent CD8^+^ T cell indices had independent prognostic significance, the complex interplay involved in tumor progression prevented any one of these factors from providing an accurate prediction of survival in patients with PDAC. Therefore, an integrated comprehensive profile must be developed to predict the survival of patients with PDAC. Based on the R software analysis, a nomogram model for OS of patients with PDAC was established using the eight independent prognostic factors (**Figure 6A**). The concordance index (C-index) values for predicting OS were 0.691 (95% CI, 0.659–0.723) and 0.712 (95% CI, 0.673–0.752) in the primary training and external validation cohorts, respectively. The above results indicated that the prediction model possessed good discriminative ability. The calibration curve for the probability of 1-, 2- and 3-year OS showed optimal agreement between the actual observed survival and the novel nomogram prediction in the training and external validation cohorts (**Figure 6B and 6C**), indicating good predictive accuracy of the nomogram model.

### Development of an integrated nomogram-based CSC-immune-TB profile showed superiority in survival prediction compared with the TNM stage model

**Figure 7:**
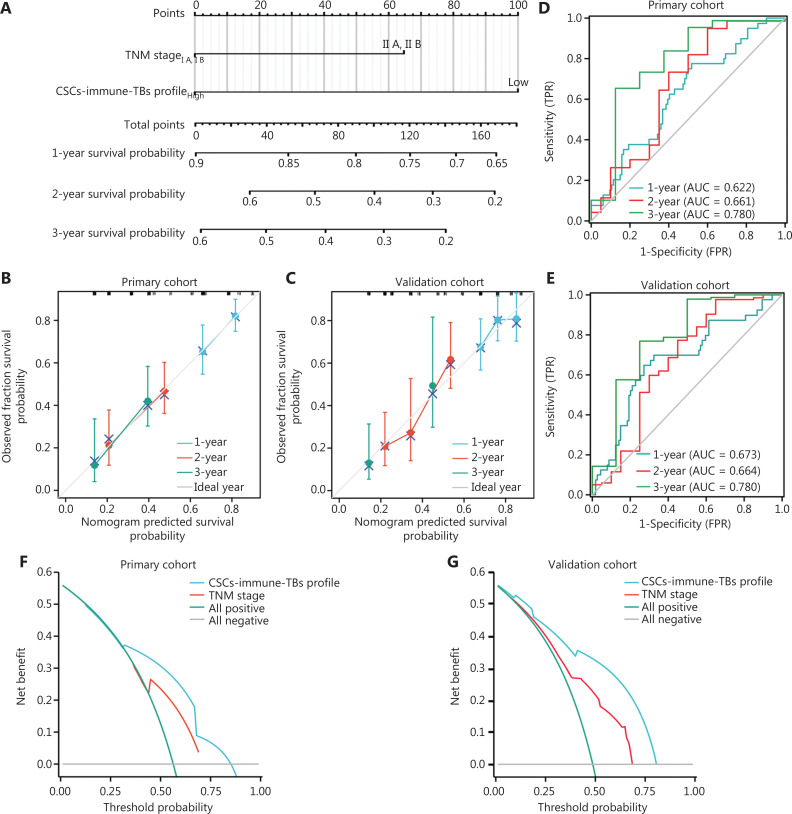
Development of an integrated nomogram-based CSC-immune-TB profile showing superiority in survival prediction compared with the TNM stage model. (A) Integrated nomogram for predicting the overall survival of patients with PDAC after surgery using the CSC-immune-TB profile model. To estimate the survival rate of an individual patient, the value of each factor was acquired on each variable axis, followed by a line drawn straight upward to determine the points. The sum of these eight numbers is located on the Total Points axis, then a line is drawn downward to the survival axis to determine the likelihood of survival. (B, C) The calibration curves for predicting patient overall survival (OS) in a retrospective primary cohort (B) and retrospective validation cohort (C) using the CSCs-immune-TB profile model. The nomogram-predicted probability of survival is plotted on the x-axis and the actual survival is plotted on the y-axis. (D, E) Time-dependent ROC curve of CSC-immune-TB scores in prediction analysis of 1-, 2-, and 3-year survival time of patients in a retrospective primary cohort (D) and retrospective validation cohort (E). The ordinate represents the sensitivity, the horizontal coordinate represents the 1-specificity, the blue curve indicates 1 year-survival time, the red curve indicates 2 year-survival time, and the green curve indicates 3 year-survival time. (F, G) Decision curve analysis (DCA) based on the predictive model of CSC-immune-TB profile and TNM stage of patients in a retrospective primary cohort (F) and retrospective validation cohort (G). The blue curve indicates CSC-immune-TB profile, the red curve indicates TNM stage, the green curve indicates all positive, and the gray curve indicates all negative.

The wide clinical application of this prognostic nomogram was hindered by the elaborate calculation process. The contributions of the CD8/CD133 and CD8/TB indices on patient survival accounted for most of the seven CSC-immune-TB-related parameters. We modified this nomogram into a simple integrated stemness-TB-immune profile system based on the point of the two indices in the nomogram (**Figure 7A**). Then, we calculated the integrated nomogram-based CSC-immune-TB profile index of each patient by summing the values of the CD8/CD133 and CD8/TB indices. The C-index values for predicting OS were 0.746 (95% CI, 0.712–0.781) and 0.755 (95% CI, 0.723–0.772) in the primary training and external validation cohorts, respectively. The above results indicated that the predictive model had better discriminative ability in the training and external validation cohorts (**Figure 7B and 7C**), indicating good predictive accuracy of the nomogram model. In addition, the sensitivity and specificity of the nomogram model evaluated via a time-dependent ROC curve analysis confirmed the good performance of survival predication for this model (**Figure 7D and 7E**). Importantly, the decision curve analysis (DCA) for OS in the primary and validated cohorts indicated that our nomogram displayed better performance than the TNM staging system (**Figure 7F and 7G**).

## Discussion

Performing comprehensive analysis of the TME components is a recent research trend. The imbalance between ‘tumor defenders’ and ‘tumor attackers’ contributes to tumor progression. AI-based automated image analysis and mIF methods have provided the possibility of analyzing the distribution of multiple TME components simultaneously. Based on this methodology, our results showed that the presence of high CD8^+^ T lymphocyte density correlated with favorable clinicopathologic features and predicted a better prognosis. Furthermore, the presence of high CD133^+^ CSCs and TB density correlated with unfavorable clinic pathologic features and predicted a poor prognosis.

Moreover, our results demonstrated that the density of CD8^+^ T lymphocytes was negatively correlated with the density of TB in patients with PDAC. Lang-Schwarz et al.^31^ found that the absence of TILs was associated with accumulating TB in patients with CRC. This finding may indicate that an impaired anti-tumor immune response niche forms during the transition of TB from epithelial-mesenchymal transition (EMT) phenotypes. In addition, the decreased expression of major histocompatibility complex (MHC) molecules and negative regulatory immune checkpoints, such as PD-L1, Tim-3, NOX2, and IDO1, during the transition of TB from EMT phenotypes may cause immune evasion and immune exhaustion^32^. Our data also showed a significant inverse relationship between the densities of CD8^+^ T lymphocytes and CD133^+^ CSCs in patients with PDAC, suggesting immune evasion is a malignant feature of CSCs. Ramgolam et al.^33^ reported that the expression of MHC molecules is down-regulated in melanoma spheroid cells, leading to an inhibition of the allogeneic immune response of T cells. Moreover, loss of CSC tumor-associated antigens leads to immune evasion^34^. As expected, we showed that fewer CD8^+^ T lymphocyte-infiltrating PDXs corresponded with increased TB or CD133^+^ CSCs. This finding indicates that TB and CSCs evade the anti-tumor host immune response and contribute to tumor progression. Considering that patients sharing similar levels of CD8^+^ T lymphocytes, CSCs, or Ts exhibited varied clinical outcomes, we are of the opinion that a comprehensive analysis of the TME must be performed to accurately predict patient outcome. Lugli et al.^30^ used an ‘anti-/pro-tumor’ approach model to predict prognosis in patients with CRC. In the present study another ‘anti-/pro-tumor’ model defined by the CD8^+^ T lymphocyte/CD133^+^ CSC and CD8^+^ T lymphocyte/TB indices was used as an independent prognosis factor in patients with PDAC. The spatial relationships of individual cellular components in patients with PDAC may offer novel insights into the dynamic and complex processed leading to PDAC progression. Specifically, we found that the percentage of CD8^+^ T cells within a 20-μm distance around the CD133^+^ CSCs or CK19^+^ TB was positively correlated with patient survival, suggesting that the CSC/TB-adjacent CD8^+^ cells may serve as a predictor of PDAC prognosis.

Most studies have focused on single component in the TME, which is dismissive of the dynamic equilibrium of different factors in the TME and do not fully illustrate the TME landscape. These indicators, including the CD8/CD133 and CD8/TB indices, as well as the CD133-adjacent CD8^+^ T and TB-adjacent CD8^+^ T cells, have been successfully established as “anti-tumor” and “pro-tumor” models. The CD8/CD133 and CD8/TB indices, as well as the CD133-adjacent CD8^+^ T and TB-adjacent CD8^+^ T cells, encompass a tumor and host-related features, and therefore represent a more advantageous ‘multi-marker’ approach to prognosis, thus better reflecting tumor dynamics in the TME. Considering the heterogeneity of the TME in patients with PDAC, these indicators still do not completely represent the comprehensive interaction among many components. Many other TME factors, such as tertiary lymphoid structures (TLSs)^35^, Tregs, MDSCs, TAMs, TANs, and (CAFs) account for PDAC progression and prognosis. Many other TME components can also be used to establish “anti-/pro-tumor” models. TAMs are normally classified into two major phenotypes (M1 and M2), in which M1 TAMs suppress cancer progression, while M2 TAMs promote cancer progression. Patients with increased intra-tumor M1/M2 TAM ratios have with an improved 5-year prognosis in ovarian cancer patients^36^. Furthermore, the ratio of alpha smooth muscle actin (αSMA)^+^ CAF/fibroblast activation protein (FAP)^+^ CAF has significant prognostic value, thus showing that patients with high αSMA and low FAP expression have significantly better OS^10^. Moreover, the neutrophil-to-lymphocyte ratio (NLR), platelet-to-lymphocyte ratio (PLR), and lymphocyte-to-monocyte ratio (LMR) are among the many surrogate biomarkers for inflammation that have been associated with outcomes in patients with gastrointestinal cancer^37^. Other “anti- /pro-tumor” models, such as the CD8/MDSC, CD8/TAM, CD8/CAF, TLS/MDSC, and TLS/TAM ratios, warrant further investigation in a corollary study. Comprehensive analysis of these components of the TME could also provide a promising approach to predict the prognosis of PDAC patients.

We also constructed a nomogram-based prediction model that includes CD8^+^ T cells, CD133^+^ CSCs, TB, CD8/CD133, CD8/TB, CD133-adjacent CD8^+^ T cells, TB-adjacent CD8^+^ T cells, and the TNM stage. A multi-parameter analysis limited the clinical application. Thus, considering the contribution of different variables on survival, we finally chose the CD8/CD133 and CD8/TB indices to re-construct an ‘immune-CSC-TB profile’ to simplify the prediction model. Furthermore, ROC curve and DCA analysis confirmed the predictive value of this model. In addition, our ‘immune-CSC-TB profile’ model was shown to be superior to the TNM stage in predicting survival of patients with PDAC. Thus, this nomogram-based prediction model provides visual representation of individual risk assessment. There were some limitations with the nomogram-based prediction model. First, the sample size used for establishment of this model was relatively small and the diagnostic performance needs to be further tested in another larger PDAC patient cohort. Second, only CD8^+^ T cells, CD133^+^ CSCs, and TB were used in this study and the TME variables were rather limited. More effective prognostic factors should be considered for inclusion in this model. Third, this research excluded unresectable tumors or distant metastases, thus our results cannot be generalized to the entire TNM staging system because the M1 stage was excluded. Fourth, this AI-based predication model exhibited complexity and high cost compared to the canonical TNM stage model. Thus, future studies are needed to evaluate many other critical immune markers for inclusion in this system and to develop more technology with relatively low cost to simplify this model.

Checkpoint blockade, such as PD-1/PD-L1 blockade, is a pillar of cancer immunotherapy for several tumor types, including melanoma, and lung, renal, and bladder cancers; however, the efficacy in patients with PDAC remains poor^38^. Increasing evidence suggests that PDAC secretes a series of immune-modulating factors that induce an immunosuppressive microenvironment composed of Treg cells, MDSCs, CAFs, and TAMs^39^, which reduce the accumulation and killing effects of cytotoxic CD8^+^ T cells and further results in an immunosuppressive environment resistant to immunotherapy. Precise assessment of the TME is critical to identify suitable immunotherapeutic options. In the current study the AI-based evaluation system facilitated determination of the immune landscape status by quantifying the different components of the immune microenvironment. For example, high CD8^+^ T cell, CD8^+^ T cell/TB, and CD8^+^ T cell/CSC indices predicted an active anti-tumor immune microenvironment, which was suitable for a checkpoint regimen, such as PD-1/PD-L1 Tim-3 blockade, and a CD40 agonist^40^. In addition, PDAC patients with a high accumulation of CSCs could be candidates for CSC-targeted therapy, such as anti-CD133 and anti-CXCR4/CXCL12 therapies^41^. Moreover, an AI-based system can also be used to evaluate other components of the TME in patients with PDAC, such as αSMA CAFs, MDSCs, and stromal-targeting therapy, such as PEGPH20 therapy and focal adhesion kinase (FAK) inhibition, and myeloid suppression therapy, such as CCR2 and CSF-1R inhibitors^42–45^. Taken together, an AI-based automatic system can be used to evaluate the immune landscape in PDAC patients to guide the choice of postoperative treatment, such as the immunotherapy, anti-CSC therapy, and stromal-targeted therapy.

## Conclusions

**Figure 8:**
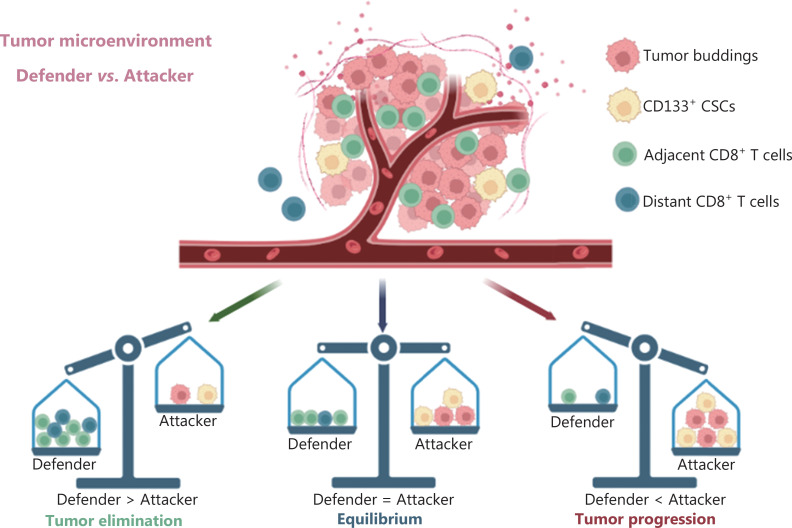
Schematic illustration for the entire study.

Taken together, the spatial relationship among CD8^+^ T cells, CSCs, and TB within the TME on a single tissue section provided novel strategies to predict prognosis of patients with PDAC through an AI-based comprehensive analysis and machine learning workflow. We built a nomogram-based immune-CSC-TB profile system based on two variables (CD8/CD133 and CD8/TB indices). This comprehensive nomogram-based prediction model can serve as an important complement to the TNM system for operable patients with PDAC. Future studies are warranted to measure other critical TME-associated markers for inclusion in the system (**Figure 8**).
