# Supplementary material for: Artificial intelligence-based comprehensive analysis of immune-stemness-tumor budding profile to predict survival of patients with pancreatic adenocarcinoma
Source: Cancer Biol Med. 2023 Mar 24;20(3):196–217. doi: 10.20892/j.issn.2095-3941.2022.0569 (PMC10038069; doi:10.20892/j.issn.2095-3941.2022.0569)
Supplement: Supplementary file 1 [file cbm-20-196-s001.pdf]

Supplementary Figures and Figure legends

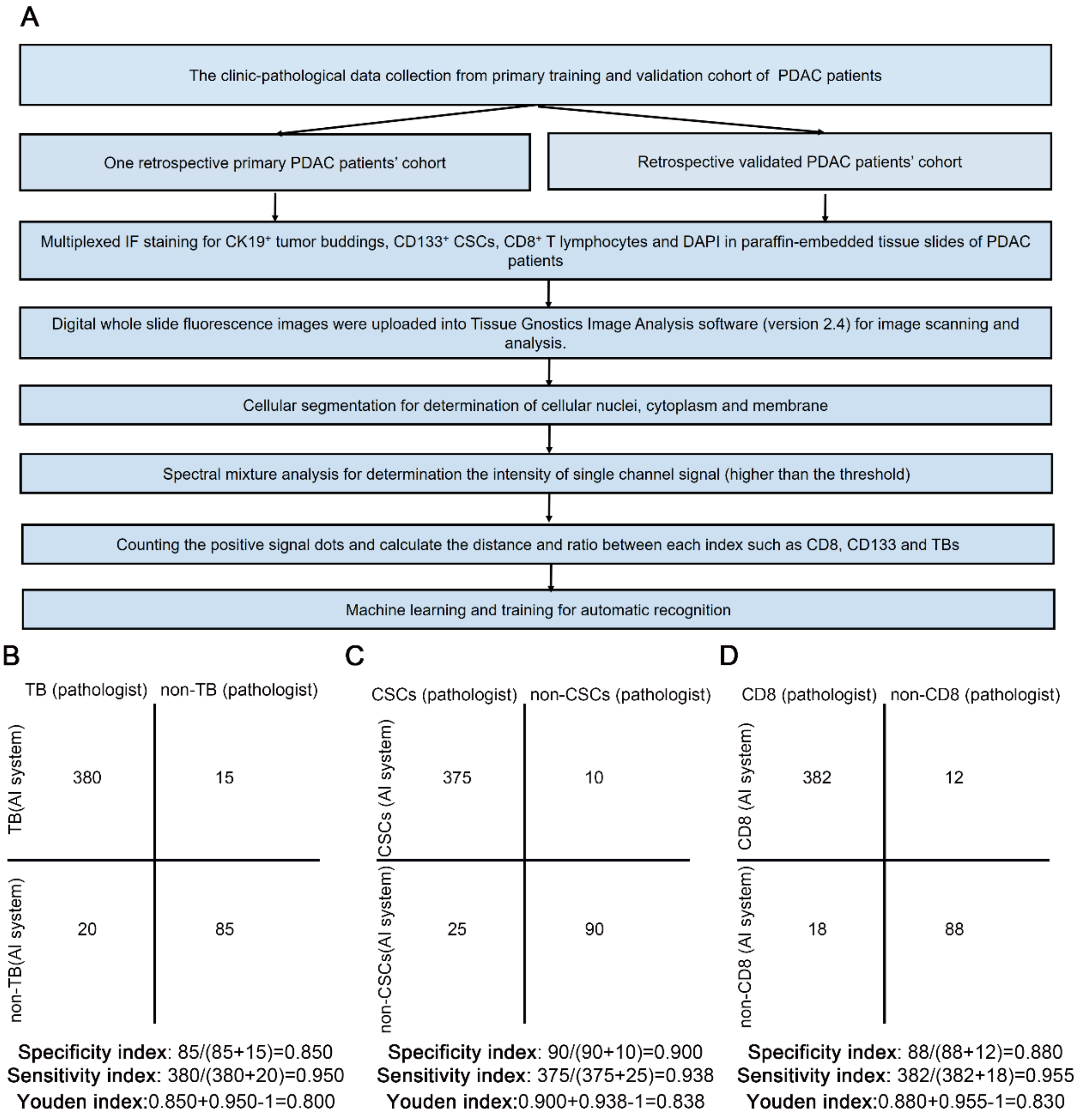

Figure S1. (A) The workflow for the machine learning and training process for automatic recognition. **(B-D).** 500 mIF images were simultaneously evaluated by AI-based automatic recognition and experienced pathologists. The results recognized by experienced pathologists were determined as "True Positive" and "True Negative". The specificity index, sensitivity index and Youden index were all analysed for the AI-based recognition system.

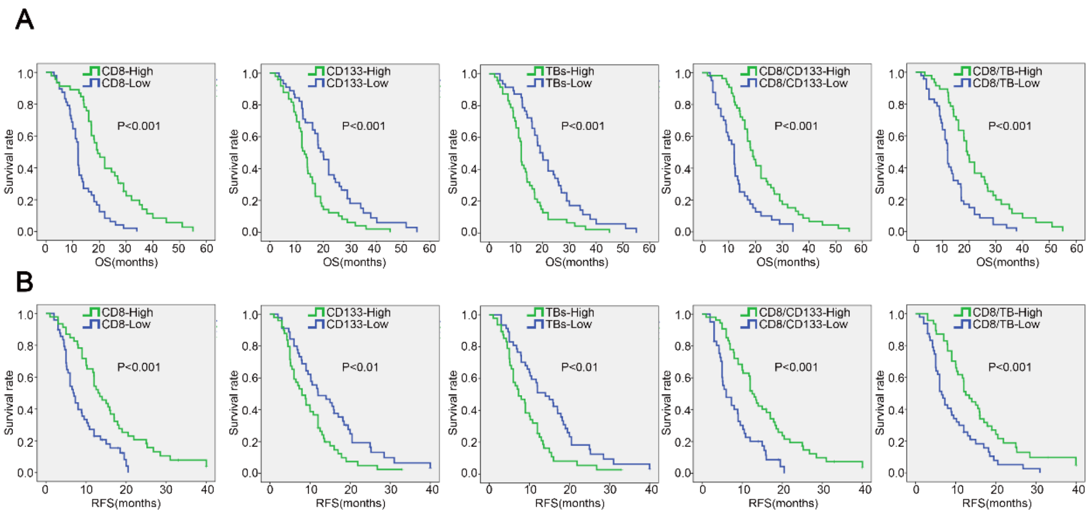

Figure S2. Correlation of CD8$^+$ T cell index, CD133$^+$ CSC index, TB index, CD8$^+$ T cell/CD133$^+$ CSC index, CD8$^+$ T cell/TB index and clinical outcome in PDAC cohort from another center (Department of Hepatopancreatobiliary Surgery, Tongliao City Hospital). (A) Correlation of CD8$^+$ T cell index, CD133$^+$ CSC index, TB index, CD8$^+$ T cell/CD133$^+$ CSC index, CD8$^+$ T cell/TB index and overall survival time in PDAC cohort. **(B)** Correlation of CD8$^+$ T cell index, CD133$^+$ CSC index, TB index, CD8$^+$ T cell/CD133$^+$ CSC index, CD8$^+$ T cell/TB index and relapse-free survival time in PDAC cohort.

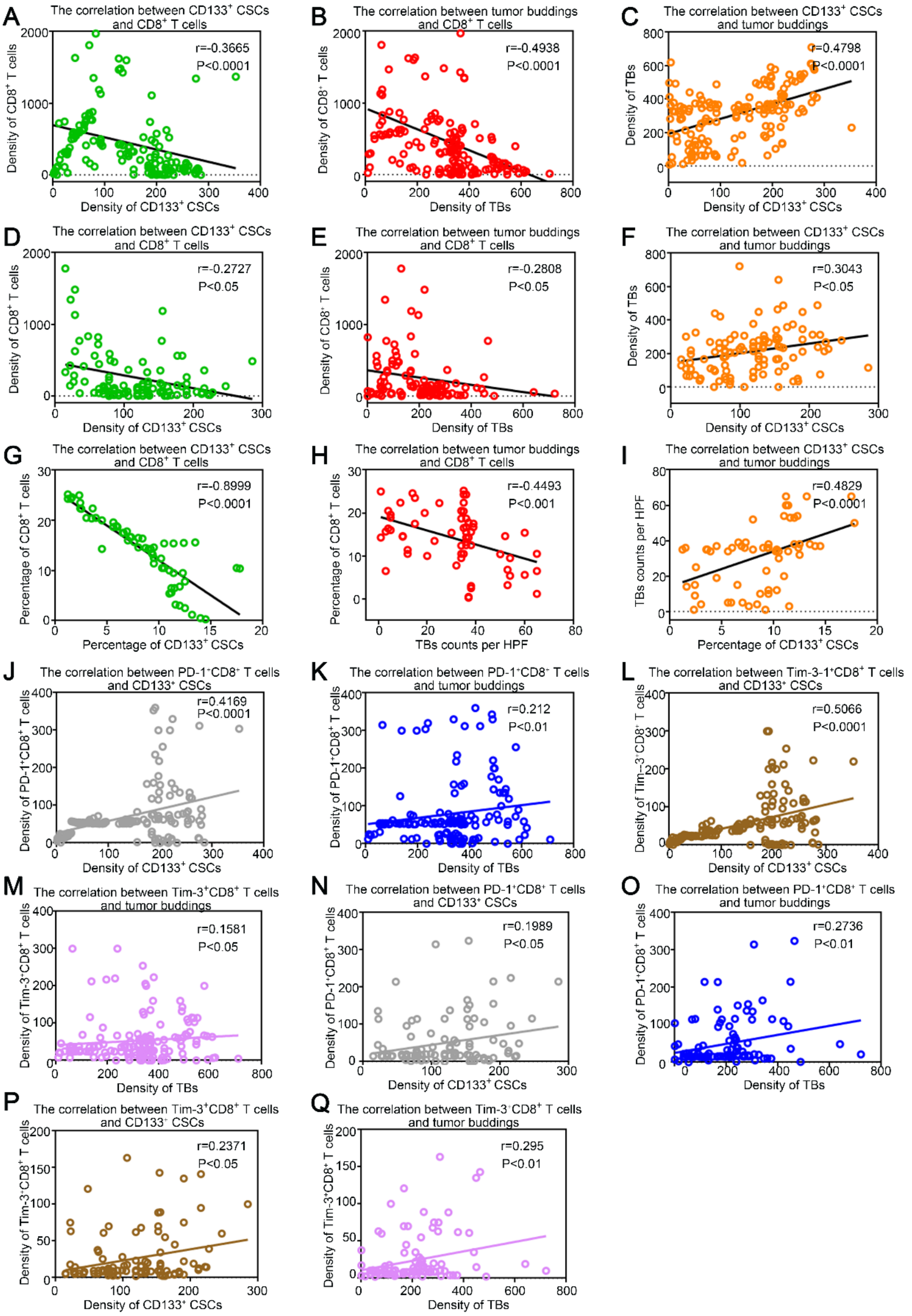

Figure S3: **Relationship between the density of tumor-infiltrating CD8+ T cells, CD133+ CSCs and tumor buddings.** (**A**) Correlation between the densities of tumor-infiltrating CD8+ T cells and CD133+ CSCs in retrospective primary training cohort. Spearman correlation analysis were performed. (**B**) Correlation between the densities of tumor-infiltrating CD8+ T cells and tumor buddings in retrospective primary training cohort. Spearman correlation analysis were performed. (**C**) Correlation between the densities of tumor-infiltrating CD133+ CSCs and tumor buddings in

retrospective primary training cohort. Spearman correlation analysis were performed. (**D**) Correlation between the densities of tumor-infiltrating CD8+ T cells and CD133+ CSCs in retrospective validated cohort. Spearman correlation analysis were performed. (**E**) Correlation between the densities of tumor-infiltrating CD8+ T cells and tumor buddings in retrospective validated cohort. Spearman correlation analysis were performed. (**F**) Correlation between the densities of tumor-infiltrating CD133+ CSCs and tumor buddings in retrospective validated cohort. Spearman correlation analysis were performed. (**G**) Correlation between the percentages of tumor-infiltrating CD8+ T cells and CD133+ CSCs in perspective validated cohort. Spearman correlation analysis were performed. (**H**) Correlation between the percentage of tumor-infiltrating CD8+ T cells and counts of tumor buddings per high power field (HPF) in perspective validated cohort. Spearman correlation analysis were performed. (**I**) Correlation between the percentage of tumor-infiltrating CD133+ CSCs and counts of tumor buddings per high power field (HPF) in perspective validated cohort. Spearman correlation analysis were performed. (**J**) Correlation between the densities of tumor-infiltrating PD-1+CD8+ T cells and CD133+ CSCs in retrospective primary training cohort. Spearman correlation analysis were performed. (**K**) Correlation between the densities of tumor-infiltrating PD-1+CD8+ T cells and tumor buddings in retrospective primary training cohort. Spearman correlation analysis were performed. (**L**) Correlation between the densities of tumor-infiltrating Tim-3+CD8+ T cells and CD133+ CSCs in retrospective primary training cohort. Spearman correlation analysis were performed. (**M**) Correlation between the densities of tumor-infiltrating Tim-3+CD8+ T cells and tumor buddings in retrospective validated cohort. Spearman correlation analysis were performed. (**N**) Correlation between the densities of tumor-infiltrating PD-1+CD8+ T cells and CD133+ CSCs in retrospective validated cohort. Spearman correlation analysis were performed. (**O**) Correlation between the densities of tumor-infiltrating PD-1+CD8+ T cells and tumor buddings in retrospective validated cohort. Spearman correlation analysis were performed. (**P**) Correlation between the densities of tumor-infiltrating Tim-3+CD8+ T cells and CD133+ CSCs in retrospective validated cohort. Spearman correlation analysis were performed. (**Q**) Correlation between the densities of tumor-infiltrating Tim-3+CD8+ T cells and tumor buddings in retrospective validated cohort. Spearman correlation analysis were performed.

A

Retrospective primary training cohort

		TB-adjacent CD8+ T cells		Spearman R	*P*
		Low	High		
CD133+CSC-adjacent CD8+ T cells	Low	65	31	0.343	0.000
	High	21	43		

B

Retrospective validation cohort

		TB-adjacent CD8+ T cells		Spearman R	*P*
		Low	High		
CD133+CSC-adjacent CD8+ T cells	Low	46	14	0.576	0.000
	High	9	39		

C

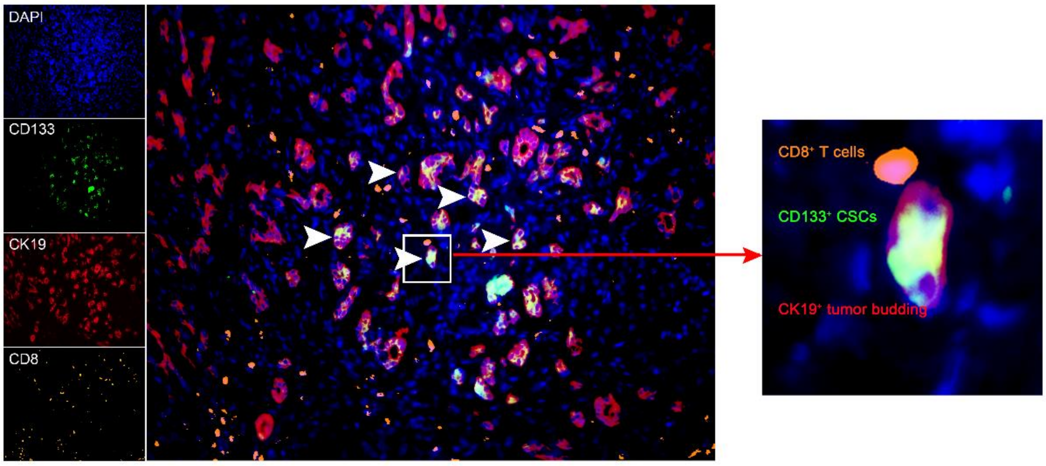

Figure S4: Relationship between the density of tumor-infiltrating CD8$^+$ T cells, CD133$^+$ CSCs and tumor buddings. (**A-B**) Correlation between the densities of TB-adjacent CD8$^+$ T cells and CD133+ CSC-adjacent CD8$^+$ T cells in retrospective primary training cohort (A) and retrospective validated cohort (B). (**C**) The representative mIF images of co-localization of CD133$^+$ CSCs, CK19$^+$ tumor buddings (TBs) and CD8$^+$ T cells.

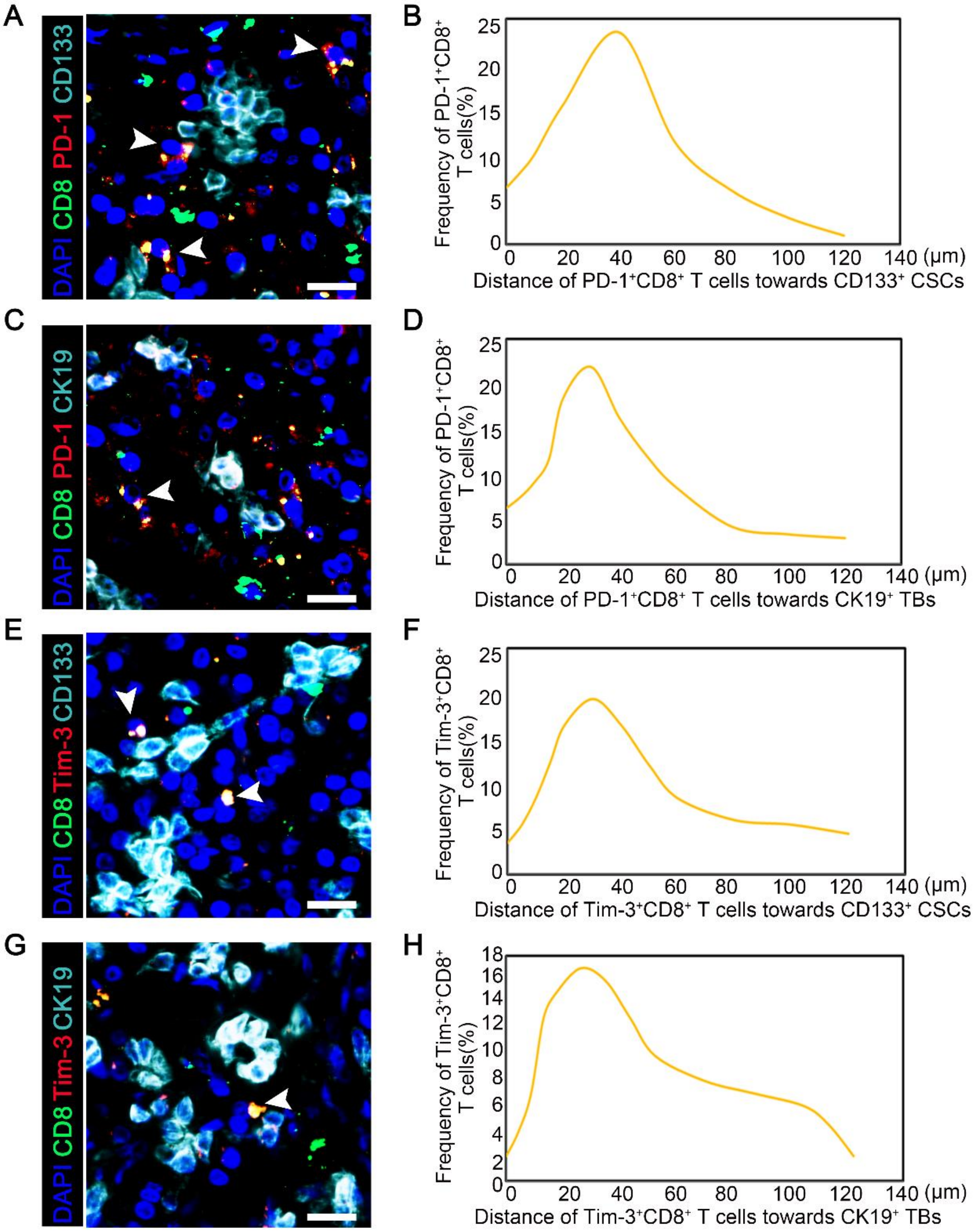

Figure S5. The spatial distribution of PD-1$^+$CD8$^+$ T cells and Tim-3$^+$CD8$^+$ T cells towards CD133$^+$ CSCs and CK19$^+$ tumor buddings. (**A-B**) Representative mIF images of PD-1$^+$CD8$^+$ T cells and CD133$^+$ CSCs (A). The frequency of PD-1$^+$CD8$^+$ T cells with different distance towards CD133$^+$ CSCs were analyzed by AI system (B). Scale bar, 50μm**.** (**C-D**) Representative mIF images of PD-1$^+$CD8$^+$ T cells and CK19$^+$ TBs (C). The frequency of PD-1$^+$CD8$^+$ T cells with different distance towards CK19$^+$ TBs were analyzed by AI system (D). Scale bar, 50μm**.** (**E-F**) Representative mIF images of Tim-3$^+$CD8$^+$ T cells and CD133$^+$ CSCs (E). The frequency of Tim-3$^+$CD8$^+$ T cells with different distance towards CD133$^+$ CSCs were analyzed by AI system (F). Scale bar, 50μm**.** (**G-H**) Representative mIF images of Tim-3$^+$CD8$^+$ T cells and CK19$^+$ TBs (G). The frequency of Tim-3$^+$CD8$^+$ T cells with different distance towards CK19$^+$ TBs were analyzed by AI system (H). Scale bar, 50μm**.**
