# Supplementary material for: Artificial intelligence-based comprehensive analysis of immune-stemness-tumor budding profile to predict survival of patients with pancreatic adenocarcinoma
Source: Cancer Biol Med. 2023 Mar 24;20(3):196–217. doi: 10.20892/j.issn.2095-3941.2022.0569 (PMC10038069; doi:10.20892/j.issn.2095-3941.2022.0569)
Supplement: Supplementary file 2 [file cbm-20-196-s002.pdf]

Supplementary Materials and Methods

Study design and patient cohort

This study was performed on two independent retrospective cohorts of patients with PDAC and another perspective cohort of patients with PDAC. The inclusion criteria were as follows: no history of other malignancies; no neo-adjuvant chemotherapy or radiotherapy; no unrespectable tumours or distant metastases pre-operation; histopathologically verified pancreatic adenocarcinoma; with complete clinic pathological data and 100% follow-up information; and systemic gemcitabine-based chemotherapy. The exclusion criteria included the following: perioperative mortality and developed a second primary cancer during follow-up. The retrospective primary training cohort included 160 patients with PDAC at the Tianjin Medical University Cancer Institute and Hospital, China from July 2011 to January 2015. 108 Patients from the Tianjin Medical University Cancer Institute and Hospital, China from July 2016 to January 2018 were assigned to another retrospective validation cohort. The perspective validation cohort included 63 patients with PDAC at the Tianjin Medical University Cancer Institute and Hospital, China from January 2016 to January 2017. Furthermore, another retrospective validated cohort including 95 PDAC patients from Department of Hepatopancreatobiliary Surgery, Tongliao City Hospital, Tongliao, Inner Mongolia, China were also recruited.

All patients were categorized in accordance with the NCCN TNM staging system. The usage of these specimens and the patient information were approved by the Ethics Committee of the Tianjin Medical University Cancer Institute and Hospital (Tianjin, China). All patients provided written consents for the use of their specimens and disease information for future investigations in accordance with the ethics committee and Declaration of Helsinki (Ethics approval ID: AE-2021021 and 2021024).

Immunohistochemistry (IHC), haematoxylin and eosin (H&E), image capture and scoring

IHC assays for CD8, CD133 and CK19 were performed on pancreatic cancer tissues in accordance with standard protocols. In Brief, at the first day, paraffin-embedded specimen slides with a thickness of 5μm were deparaffinised and then heated in a pressure plot for 2.5 min to retrieve the antigens, followed by 3% hydrogen peroxide. Subsequently, after being blocked with 1% bovine serum albumin blocking buffer, the slides were incubated with primary antibodies overnight at 4 ℃. At the second day, the sections were incubated with secondary antibodies for 30 min at an incubator at 37°C. The diaminobenzidine (DAB) substrate kit (ORIGENE, ZLI-9019) was used for the chromogenic reaction. Finally, the slides were counterstained with hematoxylin, dehydrated and cover slipped.

For the IHC and H&E assays, all specimens were assessed by two independent experienced pathologists who were blinded to the patients' clinical features and outcomes. Quantitative analyses of $CD8^+$ T cell, $CD133^+$ CSCs and $CK19^+$ TBs were performed on full slides. Five random fields (100×magnification) were evaluated under a light microscope.

Multiplexed immunofluorescence

The specificity of the antibodies was validated by IHC under a bright-field microscope, and then each target was detected by uniplex immunofluorescence. Serial sections of PDAC were subjected to uniplex immunofluorescence using opal 7-colour manual IHC kits (Perkin Elmer, 2395285) in accordance with the manufacturer's instructions. In brief, 5μm PDAC slides were deparaffinized and then rehydrated through a graded series of ethanol solutions:(100% 1×10min; 95% 1×10min; and rinse in 70%) prior to antigen retrieval in heated citric acid buffer (pH 6.0) in a microwave treatment for 15min (EZ Retriever microwave). The Slides were allowed to cool down for 20 minutes at room temperature and then washed by TBST. TBST was used for all washing steps. Tissues were then blocked with blocking/dilution buffer for 10 min. The slides were incubated for 2 h with the same antibodies as those for IHC at these dilutions: CD8 (dilution 1:100), CD133 (dilution 1：100) and CK19 (dilution 1:100). The slides were then washed three times for 3min each. The slides that had previously been incubated with CD8 antibodies were then incubated in pre-diluted anti-rabbit/mouse horseradish peroxidase-conjugated (HRP)-conjugated secondary antibodies for 10 min. Following three more washes of 3 min, the slides were incubated for 10 min with opal 520 for anti-CD8 antibody visualization at 1:100 dilution. The slides that had been previously incubated with anti-CD133 antibody were incubated in opal 570 in an antibody diluent at 1:100 dilution for 10 minutes. The slides that had been previously incubated with anti-CK19 antibody was incubated in opal 690 in an antibody diluent at 1:100 dilution for 10 min. Finally, following a washing step, the slides were counterstained for 10 min with DAPI at 1:20 dilution in deionized water. Optimal antibody dilutions in the uniplexed immunofluorescence slides were selected to ensure optimal signal-to-noise ratio and specific staining pattern when digitized on the Tissue Gnostics whole slide scanner (Zeiss). Fluorescence slides were compared with serial sections of bright-field DAB slides using the same antibodies. Positive and negative controls were included in each staining run. The positive controls for CD8 antibodies were the spleen as specified above.

After determination of the appropriate dilution of antibodies and immunofluorescence signals, we assessed them multiplexed on a single tissue section. In brief, 5μm PDAC slides were deparaffinized and rehydrated through a graded series of ethanol solutions:(100% 1×10min; 95% 1×10min; and rinse in 70%) prior to antigen retrieval in heated citric acid buffer (pH 6.0) in a microwave treatment for 15min (EZ Retriever microwave). The first run of labelling included the primary antibody against

CD8 prior to antibody stripping, followed by labelling with primary antibodies against CD133 and CK19 prior to counterstaining with DAPI. Our multiplexed sections showed no dramatic change in specific signals or nonspecific background signals compared with the uniplex-labelled serial sections. The stained slides were scanned over the whole slide using the Tissue Gnostics system (Zeiss). A slide reviewer was used to select the regions of interest in an unbiased manner. The exposure times for DAPI, opal 520, opal 570, and opal 690 channels were 10, 20, 25, and 100ms, respectively. The selected images were then captured with a ×20 lens and unstained tissues were used to extract the tissue auto-fluorescence. Images were analyzed using Tissue Gnostics analysis software 2.4.

Artificial intelligence (AI)-based image analysis

Digital whole slide fluorescence images were uploaded into Tissue Gnostics Image Analysis software (version 2.4) for image analysis. Cellular segmentation models achieve the goal of pixel-wise binary classification based on setting intensity thresholds for an image and using the min and max thresholds to classify the pixels in an image as cell objects or background. Before we assessed multiplexed IHC on a single tissue section, the slides staining with single primary antibody and related secondary fluorophores were analyzed and the fluorescence signal in each channel were acquired. Moreover, the blank slide with no any primary antibody staining were also analyzed and the background auto-fluorescence of each channel were also acquired. By comparing the difference of the auto-fluorescence intensity of the blank slide and the fluorescence intensity of the slides stained with single primary antibody and related fluorophores, we finally determined the optimal threshold to distinguish the auto-fluorescence signal and the positive-fluorescence signal. Full algorithm workflow and settings are shown as below:

Cellular segmentation

The nuclei in the whole slide image were automatically segmented using Tissue Gnostics analysis software 2.4. We determined the optimal module parameters for nuclei detection, such as different dye weights (DAPI nuclear weight=5, opal 520 nucleus weigh=0.15, opal 570 nucleus weight=0.243, opal 690 nucleus weight=0.113, nuclear contrast threshold (0.4), minimum nuclear intensity (0.032), nuclear segmentation aggressiveness (0.75), and default nuclear size setting (1–450 μm^2). All samples were analyzed using these specific settings. After determination the position of nuclei, the cytoplasm were segmented using the default cytoplasm size setting (450μm^2-1000μm^2).

Recognition and analysis of tumor-infiltrating $CD8^+$ T lymphocytes

After segmenting the nuclei (DAPI) under the same analysis module, the cells were then classified as CD8 (opal 520) positive based on dye nucleus-positive threshold

(opal 520 0.344), cytoplasm-positive threshold (opal 520 0.025) and membrane-positive threshold (opal 520 0.025). Consistent settings of analysis algorithm were applied to all slides of patients. The algorithm was used to automatically quantify the number of each lymphocyte classification across the whole slide image. The area of analyzed fields was calculated, and the density of $CD8^+$ T lymphocytes (number/area) was acquired.

Recognition and analysis of tumor-infiltrating $CD133^+$ T lymphocytes

After segmenting the nuclei (DAPI) under the same analysis module, the cells were classified as CD133 (opal 570) positive based on dye nucleus-positive threshold (opal 570 0.524), cytoplasm-positive threshold (opal 570 0.045) and membrane-positive threshold (opal 570 0.045). Consistent settings of analysis algorithm were applied to all slides of patients. The algorithm was used to automatically quantify the number of each $CD133^+$ cell classification across the whole slide image. The area of analyzed fields was calculated and the density of $CD133^+$ CSCs (number/area) were acquired.

Recognition and analysis of tumor-infiltrating $CK19^+$ TBs and $CK19^+$ PDAC ducts

After segmenting the nuclei (DAPI) under the same analysis module, the cells were then classified as CK19 (opal 690) positive based on dye nucleus-positive threshold (opal 690 0.624), cytoplasm-positive threshold (opal 690 0.025) and membrane-positive threshold (opal 690 0.025). Consistent settings of analysis algorithm were applied to all slides of patients. A TB is typically defined as a single tumor cell or tumor cell cluster of up to five cells at the invasive tumor front. Thus, once tumor regions were detected, nuclei were segmented using the methodology described above and cancer cells were classified based on CK19 positivity in the nucleus (0.624), cytoplasm (0.025), and membrane (0.025). Then, $CK19^+$ TB and $CK19^+$ PDAC ducts were recognized according to the number of nuclei and morphology. The density (number/mm^2) and number of TBs were then exported from the algorithm.

Spatial analysis of TBs, CSCs and $CD8^+$ T cells

Spatial distributions of the TBs, CSCs and $CD8^+$ T cells were imported into a spatial dot plot within the Tissue Gnostics software. According to this plot, the spatial analysis algorithm was utilized to calculate the number of $CD8^+$ T cells between 0 and 100 μm radii of TBs or CSCs in consecutively increasing 20 μm steps, creating five classes: 0–20 μm, 20–40 μm, 40–60 μm, and so on. Finally, the number and densities of $CD8^+$ T cells within these distances were established.

Establishment of patient-derived xenograft (PDX) and culture

Fresh PDAC tumor tissues were mechanically sectioned into small pieces (approximately 1mm^3) and washed three times with cold phosphate-buffered saline (PBS) supplemented with 10% penicillin and streptomycin. For tumor implantation, 6-8-week-old NSG humanized mice were purchased. All mice were maintained in specific pathogen–free conditions, and animal experiment procedures were approved by the Ethics Committee of Tianjin Medical University Cancer Institute and Hospital in compliance with the principles and procedures of the NIH Guide for the Care and Use of Laboratory Animals. The mice were anesthetized, and a small incision was made on the dorsal flank. Then, tumor tissue pieces were subcutaneously implanted into the dorsal flank of each mouse, and the incision was closed. The presence of pancreatic adenocarcinoma in xenograft tumors was validated through H&E staining. When the tumour size reached approximately 1.5cm, the implanted tumors were passaged into other mice.

Flow cytometry

The relationship among CD8$^+$ T lymphocyte, CD133$^+$CSCs and TBs in PDAC tissues was investigated. Fresh PDAC specimens and PDX specimens were collected and divided into two parts, one part was immediately fixed in formalin buffer, embedded in paraffin and then prepared for detection of TBs via IHC staining. The second part was immediately digested into the single cell suspension with 1 mg/ml collagenase (Sigma-Aldrich, C2799), 2.5 U/ml hyaluronidase (Sigma-Aldrich, H3506) and 0.1 mg/ml DNase (Sigma-Aldrich DN25). The single cell suspension was stained with anti-CD8 (Biolegend, 300922) and anti-CD133 (Biolegend, 372806) antibodies. Isotype controls were used as negative controls. The data were analyzed using soft Flow Jo 10.0

Statistical analysis

Statistical analyses were performed using IBM SPSS Statistics, Version 21.0. The cut-off values of each index were determined according to their median values in the PDAC patients' cohorts. Categorical variables were compared using the $\chi 2$ test. Continuous variables were managed using the t test. The correlation between the IHC scoring and IF counting results was estimated using a Pearson coefficient analysis. Survival curves were estimated with the Kaplan-Meier method and compared using the log-rank test. Univariate and multivariate Cox analyses were performed to determine the independent risk characteristics. The hazard ratios (HRs) and 95% confidence intervals (CIs) of these variables were estimated to quantify the strength of these associations. The nomogram, calibration curve, time-dependent receiver operating characteristic (ROC) curve and decision curve analyses were conducted with R software. All statistical tests were 2-tailed. Statistical significant was considered at $P<0.05$.
