# Supplementary material for: Artificial intelligence-based comprehensive analysis of immune-stemness-tumor budding profile to predict survival of patients with pancreatic adenocarcinoma
Source: Cancer Biol Med. 2023 Mar 24;20(3):196–217. doi: 10.20892/j.issn.2095-3941.2022.0569 (PMC10038069; doi:10.20892/j.issn.2095-3941.2022.0569)
Supplement: Supplementary file 3 [file cbm-20-196-s003.pdf]

Supplementary Table1: Characteristics of PDAC patients in primary retrospective training cohort (N=160) and validation cohort (N=108) in our center

Primary cohort(N=160)		Validation cohort(N=108)	
**Clinico-pathological features**	**Frequency N(%)**	**Clinico-pathological features**	**Frequency N(%)**
**Gender**		**Gender**	
Male	66(41.2%)	Male	69(63.8%)
Female	94(58.8%)	Female	39(36.2%)
**Age**		**Age**	
≤60	79(49.3%)	≤60	64(59.2%)
＞60	81(50.7%)	＞60	44(40.8%)
**Tumor size**		**Tumor size**	
≤3.5cm	81(50.6%)	≤3.5cm	37(34.2%)
＞3.5cm	79(49.4%)	＞3.5cm	71(65.8%)
**Histological Grade**		**Histological Grade**	
G1,G2	66(41.2%)	G1,G2	53(49.1%)
G3	94(58.8%)	G3	55(50.1%)
**LN metastasis**		**LN metastasis**	
N0	64(40.0%)	N0	50(46.3%)
N1	96(60.0%)	N1	58(53.7%)
**TNM stage**		**TNM stage**	
IA,IB	75(46.9%)	IA,IB	54(50.0%)
IIA,IIB	85(53.1%)	IIA,IIB	54(50.0%)
**Nerve invasion**		**Nerve invasion**	
Yes	52(32.5%)	Yes	75(69.4%)
NO	108(67.5%)	NO	33(30.6%)
**CA19-9 (U/ml)**		**CA19-9 (U/ml)**	
≤573.3	78(48.8%)	≤573.3	64(59.3%)
＞573.3	82(51.2%)	＞573.3	44(40.7%)

Supplementary Table2: Characteristics of PDAC patients in perspective validation cohort in our center

Clinico-pathological features	Validation cohort(N=63) Frequency N(%)
**Gender**	
Male	35(55.6%)
Female	28(44.4%)
**Age**	
≤60	29(46.0%)
＞60	34(54.0%)
**Tumor size**	
≤3.5cm	32(50.8%)
＞3.5cm	31(49.2%)
**Histological Grade**	
G1,G2	28(44.4%)
G3	35(55.6%)
**LN metastasis**	
N0	33(52.4%)
N1	30(47.6%)
**TNM stage**	
IA,IB	25(39.7%)
IIA,IIB	38(60.3%)
**Nerve invasion**	
Yes	42(66.7%)
NO	21(33.3%)

Supplementary Table3: Characteristics of PDAC patients in another retrospective validation cohort from Department of Hepatopancreatobiliary Surgery, Tongliao City Hospital

Validation	cohort(N=95)
**Clinico-pathological features**	**Frequency N(%)**
**Gender**	
Male	48(50.5%)
Female	47(49.5%)
**Age**	
≤60	45(47.4%)
＞60	50(52.6%)
**Tumor size**	
≤3.5cm	35(36.8%)
＞3.5cm	60(63.2%)
**Histological Grade**	
G1,G2	73(76.8%)
G3	22(23.2%)
**LN metastasis**	
N0	58(61.1%)
N1	37(38.9%)
**TNM stage**	
IA,IB	52(54.7%)
IIA,IIB	43(45.3%)
**Nerve invasion**	
Yes	31(32.6%)
NO	64(67.4%)

Supplementary Table 4: The association of CD8 index, CD133 index, TBs index, CD8/CD133 index, CD8/TBs index with clinico-pathological features-primary retrospective cohort (N=160) in our center.

	**CD8 index**					**CD133 index**					**TBs index**					**CD8/CD133 index**					**CD8/TBs index**				
**Clinico-pathological features**	Low	high	χ2	Spearman r	P-value	Low	high	χ2	Spearman r	P-value	Low	high	χ2	Spearman r	P-value	Low	high	χ2	Spearman r	P-value	Low	high	χ2	Spearman r	P-value
**Gender**																									
Male	37	29	0.197	-0.035	0.66	36	30	0.029	0.013	0.867	24	42	0.846	-0.073	0.361	36	30	0.591	-0.061	0.445	53	13	2.071	0.114	0.152
Female	56	38				50	44				41	53				57	37				66	28			
**Age**																									
≤60	43	36	0.875	-0.074	0.353	40	39	0.610	-0.062	0.438	37	42	2.495	0.125	0.116	43	36	0.875	-0.074	0.353	57	22	0.405	-0.050	0.528
＞60	50	31				46	35				28	53				50	31				62	19			
**Tumor size**																									
≤3.5cm3	30	51	29.973	-0.433	**0.000**	52	29	7.203	0.212	**0.007**	42	39	8.572	0.231	**0.003**	34	47	17.579	-0.331	**0.000**	48	33	19.667	-0.351	**0.000**
＞3.5cm3	63	16				34	45				23	56				59	20				71	8			
**Histological Grade**																									
G1,G2	24	44	25.326	-0.398	**0.000**	45	23	7.346	0.214	**0.007**	41	27	18.968	0.344	**0.000**	26	42	19.221	-0.347	**0.000**	37	31	24.729	-0.393	**0.000**
G3	69	23				41	51				24	68				67	25				82	10			
**LN metastasis**																									
N0	19	45	35.440	-0.471	**0.000**	43	21	7.748	0.220	**0.005**	34	30	6.910	0.208	**0.008**	21	43	28.079	-0.419	**0.000**	34	30	25.273	-0.397	**0.000**
N1	74	22				43	53				31	65				72	24				85	11			
**TNM stage**																									
IA,IB	26	49	31.918	-0.447	**0.000**	53	22	16.252	0.319	**0.000**	43	32	16.639	0.320	**0.000**	30	45	19.055	-0.345	**0.000**	42	33	25.011	-0.395	**0.000**
IIA,IIB	67	18				33	52				22	63				63	22				77	8			
**Nerve invasion**																									
Yes	30	22	0.006	-0.006	**0.939**	25	27	0.997	-0.079	0.321	20	32	0.149	-0.031	0.701	31	21	0.070	0.021	0.792	40	12	0.262	-0.041	0.611
NO	63	45				61	47				45	63				62	46				79	29			
**CA19-9 (U/ml)**																									
≤573.3	31	47	21.127	-0.363	**0.000**	52	26	10.214	0.253	**0.000**	38	40	4.133	0.161	**0.042**	34	44	13.211	-0.287	**0.000**	49	29	10.661	-0.258	**0.001**
＞573.3	62	20				34	48				27	55				59	23				70	12			

Supplementary Table 5: The univariate analysis and multivariate analysis for overall survival and relapse free survival in the primary cohort (N=160) in our center

	Overall Survival						Relapse Free Survival					
	Univariate analysis			Multivariate analysis			Univariate analysis			Multivariate analysis		
**Clinico-pathological features**	HR	95% CI	P-value	HR	95%CI	P-value	HR	95% CI	P-value	HR	95%CI	P-value
**Gender**												
Male	1						1					
Female	1.391	0.972-1.990	0.071				0.630	0.401-0.989	0.352			
**Age**												
≤60	1						1					
>60	1.305	0.921-1.849	0.134				1.575	0.995-2.494	0.053			
**Tumor size**												
≤3.5cm	1						1					
>3.5cm	1.525	1.076-2.162	**0.018**				1.784	1.124-2.831	**0.014**			
**Histological Grade**												
G1,G2	1						1					
G3	1.719	1.205-2.453	**0.003**				1.396	0.891-2.186	**0.046**			
**LN metastasis**												
N0	1						1					
N1	1.814	1.270-2.590	**0.001**				1.375	0.867-2.180	**0.017**			
**TNM stage**												
IA,IB	1			1			1			1		
IIA,IIB	3.097	2.101-4.565	**0.000**	1.148	0.549-1.408	**0.045**	3.734	2.274-6.133	**0.000**	2.779	1.633-4.730	**0.000**
**Nerve invasion**												
NO	1						1					
yes	0.957	0.659-1.388	0.815				0.998	0.602-1.655	0.994			
**CA19-9 (U/ml)**												
≤573.3	1						1					
>573.3	2.111	1.456-3.061	**0.000**				1.671	1.057-2.643	**0.028**			
**CD8 index**												
low	1			1			1			1		
high	0.344	0.233-0.508	**0.000**	0.384	0.193-0.762	**0.006**	0.252	0.148-0.430	**0.000**	0.471	0.204-1.086	**0.047**
**CD133 index**												
low	1			1			1			1		
high	2.082	1.452-2.985	**0.000**	1.125	0.715-1.769	**0.016**	3.155	1.943-5.121	**0.000**	1.546	0.841-2.841	**0.016**
**TBs index**												
low	1			1			1			1		
high	2.634	1.805-3.844	**0.000**	1.286	0.679-1.737	**0.037**	6.230	3.445-11.268	**0.000**	2.967	1.491-5.902	**0.002**
**CD8/CD133 index**												
low	1			1			1			1		
high	0.300	0.203-0.443	**0.000**	0.917	0.493-1.706	**0.048**	0.148	0.084-0.261	**0.000**	0.210	0.109-0.407	**0.000**
**CD8/TBs index**												
low	1			1			1			1		
high	0.280	0.181-0.431	**0.000**	0.438	0.239-0.801	**0.007**	0.199	0.102-0.388	**0.000**	0.365	0.178-0.749	**0.006**

Supplementary Table 6: The association of CD8 index, CD133 index, TBs index, CD8/CD133 index, CD8/TBs index with clinico-pathological features- retrospective validation cohort (N=108) in our center

	**CD8 index**					**CD133 index**					**TBs index**					**CD8/CD133 index**					**CD8/TBs index**				
**Clinico-pathological features**	Low	high	χ2	Spearman r	P-value	Low	high	χ2	Spearman r	P-value	Low	high	χ2	Spearman r	P-value	Low	high	χ2	Spearman r	P-value	Low	high	χ2	Spearman r	P-value
**Gender**																									
Male	49	20	0.222	0.045	0.641	31	38	0.015	-0.012	0.903	28	41	0.317	-0.054	0.578	44	25	0.001	-0.003	0.973	37	32	0.289	-0.052	0.595
Female	26	13				18	21				18	21				25	14				23	16			
**Age**																									
⩽60	44	20	0.036	-0.018	0.852	28	36	0.166	-0.039	0.687	28	36	0.086	0.028	0.772	42	22	0.205	0.044	0.654	33	31	1.014	-0.097	0.318
＞60	31	13				21	23				18	26				27	17				27	17			
**Tumor size**																									
⩽3.5cm3	10	27	47.723	-0.665	**0.000**	23	14	6.403	0.243	**0.011**	25	12	14.357	0.365	**0.000**	13	24	20.169	-0.432	**0.000**	10	27	18.552	-0.414	**0.000**
＞3.5cm3	65	6				26	45				21	50				56	15				50	21			
**Histological Grade**																									
G1,G2	25	28	24.335	-0.475	**0.000**	36	17	21.359	0.445	**0.000**	29	24	6.257	0.241	**0.012**	26	27	9.924	-0.303	**0.001**	18	35	19.653	-0.427	**0.000**
G3	50	5				13	42				17	38				43	12				42	13			
**LN metastasis**																									
N0	29	21	5.474	-0.231	**0.016**	30	20	8.040	0.273	**0.004**	32	18	17.450	0.402	**0.000**	25	25	7.785	-0.268	**0.005**	18	32	14.420	-0.365	**0.000**
N1	46	12				19	39				14	44				44	14				42	16			
**TNM stage**																									
IA,IB	29	25	12.611	-0.342	**0.000**	39	15	31.418	0.539	**0.000**	29	25	5.453	0.225	**0.019**	25	29	14.488	-0.366	**0.000**	18	36	21.600	-0.447	**0.000**
IIA,IIB	46	8				10	44				17	37				44	10				42	12			
**Nerve invasion**																									
Yes	52	23	0.001	-0.004	0.970	38	37	2.778	0.160	0.097	32	43	0.001	0.002	0.981	48	27	0.001	0.003	0.971	41	34	0.079	-0.027	0.782
NO	23	10				11	22				14	19				21	12				19	14			
**CA19-9 (U/ml)**																									
⩽573.3	39	25	5.358	-0.223	**0.021**	33	31	2.430	0.150	**0.001**	39	25	21.621	0.447	**0.000**	35	29	5.765	-0.231	**0.016**	30	34	4.794	-0.211	**0.029**
＞573.3	36	8				16	28				7	37				34	10				30	14			

Supplementary Table 7: The univariate analysis and multivariate analysis for overall survival and relapse free survival in the validation cohort (N=108) in our center

Clinico-pathological features	Overall Survival						Relapse Free Survival					
	Univariate analysis			Multivariate analysis			Univariate analysis			Multivariate analysis		
	**HR**	**95% CI**	**P-value**	**HR**	**95%CI**	**P-value**	**HR**	**95% CI**	**P-value**	**HR**	**95%CI**	**P-value**
**Gender**												
Male	1						1					
Female	1.143	0.763-1.712	0.517				0.983	0.613-1.577	0.944			
**Age**												
≤60	1						1					
＞60	0.835	0.560-1.245	0.375				0.738	0.462-1.178	0.202			
**Tumor size**												
≤3.5cm	1						1					
＞3.5cm	1.800	1.447-3.344	**0.000**				1.616	0.807-3.237	**0.015**			
**Histological Grade**												
G1,G2	1						1					
G3	1.532	1.038-2.262	**0.032**				1.634	1.033-2.582	**0.036**			
**LN metastasis**												
N0	1						1					
N1	1.570	1.059-2.328	**0.025**				1.577	0.990-2.511	**0.050**			
**TNM stage**												
IA,IB	1			1			1			1		
IIA,IIB	2.727	1.823-4.079	**0.000**	1.921	1.253-2.945	**0.045**	3.065	1.882-4.994	**0.000**	2.503	1.501-4.171	**0.000**
**Nerve invasion**												
NO	1						1					
yes	1.196	0.783-1.826	0.407				1.157	0.711-1.884	**0.558**			
**CA19-9 (U/ml)**												
≤573.3	1						1					
＞573.3	1.454	0.976-2.165	**0.046**				1.47	0.930-2.324	**0.010**			
**CD8 index**												
low	1			1			1			1		
high	0.488	0.317-0.749	**0.001**	0.532	0.334-0.845	**0.018**	0.435	0.256-0.740	**0.002**	0.665	0.480-2.463	**0.049**
**CD133 index**												
low	1			1			1			1		
high	1.858	1.248-2.766	**0.002**	1.412	1.101-2.081	**0.022**	2.427	1.513-3.892	**0.000**	1.564	0.606-2.237	**0.039**
**TBs index**												
low	1			1			1			1		
high	1.854	1.239-2.774	**0.003**	1.208	0.939-2.383	**0.041**	2.114	1.296-3.449	**0.003**	1.647	0.842-3.221	**0.046**
**CD8/CD133 index**												
low	1			1			1			1		
high	0.223	0.137-0.363	**0.000**	0.356	0.200-0.635	**0.033**	0.452	0.275-0.742	**0.002**	0.632	0.335-1.191	**0.017**
**CD8/TBs index**												
low	1			1			1			1		
high	0.272	0.175-0.422	**0.000**	0.498	0.296-0.839	**0.041**	0.560	0.350-0.894	**0.015**	0.724	0.689-1.297	**0.044**

Supplementary table8: Detailed information of all the experimental reagents and antibodies

Reagent	Source	Catalog number
The DAB substrate kit	ORIGENE	ZLI-9019
The opal 7-color manual IHC kits	Perkin Elmer	2395285
Anti-Rabbit/mouse polymer HRP	MXB Biotechnologies	KIT-5020
Hematoxylin-Eosin (HE) Stain Kit	Solarbio	G1120
DAPI solution	Solarbio	C0065
Collagenase	Sigma-Aldrich	C2799
Hyaluronidase	Sigma-Aldrich	H3506
DNase	Sigma-Aldrich	DN25
Anti-human CD8 (IHC)	ZSGB-BIO	ZA-0508
Anti-human CD133(IHC)	Abcam	Ab226355
Anti-human CK19 (IHC)	Abcam	Ab7755
Anti-human PD-1 (IHC)	Abcam	Ab52587
Anti-human Tim-3 (IHC)	Abcam	Ab241332
Anti-human CD133 (FCM)	Biolegend	372806
Anti-human CD8 (FCM)	Biolegend	300922
